# The Oncolytic Activity of Zika Viral Therapy in Human Neuroblastoma *In Vivo* Models Confers a Major Survival Advantage in a CD24-dependent Manner

**DOI:** 10.1158/2767-9764.CRC-23-0221

**Published:** 2024-01-09

**Authors:** Joseph Mazar, Jeanne K. Brooks, Matthew Peloquin, Rosa Rosario, Emma Sutton, Matthew Longo, Dennis Drehner, Tamarah J. Westmoreland

**Affiliations:** 1 Nemours Children's Hospital, Nemours Parkway, Orlando, Florida.; 2 Burnett School of Biological Sciences, The University of Central Florida College of Medicine, Orlando, Florida.

## Abstract

Neuroblastoma is the most common extracranial tumor, accounting for 15% of all childhood cancer-related deaths. The long-term survival of patients with high-risk tumors is less than 40%, and *MYCN* amplification is one of the most common indicators of poor outcomes. Zika virus (ZIKV) is a mosquito-borne flavivirus associated with mild constitutional symptoms outside the fetal period. Our published data showed that high-risk and recurrent neuroblastoma cells are permissive to ZIKV infection, resulting in cell type–specific lysis. In this study, we assessed the efficacy of ZIKV as an oncolytic treatment for high-risk neuroblastoma using *in vivo* tumor models. Utilizing both MYCN-amplified and non-amplified models, we demonstrated that the application of ZIKV had a rapid tumoricidal effect. This led to a nearly total loss of the tumor mass without evidence of recurrence, offering a robust survival advantage to the host. Detection of the viral NS1 protein within the tumors confirmed that a permissive infection preceded tissue necrosis. Despite robust titers within the tumor, viral shedding to the host was poor and diminished rapidly, correlating with no detectable side effects to the murine host. Assessments from both primary pretreatment and recurrent posttreatment isolates confirmed that permissive sensitivity to ZIKV killing was dependent on the expression of CD24, which was highly expressed in neuroblastomas and conferred a proliferative advantage to tumor growth. Exploiting this viral sensitivity to CD24 offers the possibility of its use as a prognostic target for a broad population of expressing cancers, many of which have shown resistance to current clinical therapies.

**Significance:**

Sensitivity to the tumoricidal effect of ZIKV on high-risk neuroblastoma tumors is dependent on CD24 expression, offering a prognostic marker for this oncolytic therapy in an extensive array of CD24-expressing cancers.

## Introduction

Neuroblastoma is a childhood cancer that commonly develops along the sympathetic nervous system and adrenal glands (1). It is the most common extracranial solid tumor and the most commonly diagnosed malignancy in infants (2). Accounting for only 6% of all childhood cancers, it is responsible for a disproportionally high percentage (15%) of all childhood cancer-related deaths in the United States (3–5). The most common treatments for neuroblastoma include surgery, radiotherapy, and chemotherapy (6, 7). However, these have shown limited efficacy in as much as 60%–70% of patients with high-risk neuroblastoma (8) with common complications, such as retardation of growth, renal impairment, hearing loss, vertebral deformity, and thyroid dysfunction (9, 10). Although new therapeutic techniques exist for patients with recurrent and refractory neuroblastoma, they have the disadvantages of toxic side effects, induction of early resistance, and low specificity (11–14), leaving the long-term survival of patients with high-risk tumors at less than 40% (15, 16). Complex heterogeneity exists within the tumor subpopulation of neuroblastoma, with prognostic factors for survival including age at diagnosis, tumor site, tumor grade, histology, and amplification of the v-myc avian myelocytomatosis viral oncogene neuroblastoma-derived homolog *(MYCN)* gene. *MYCN* amplification accounts for nearly 25% of patients and is one of the most significant biomarkers for high-risk neuroblastoma, correlating with both disease advancement and poor survival (17, 18). Over the last 20 years, there has been little improvement in the overall survival of children with MYCN-amplified neuroblastoma.

Oncolytic therapies have emerged as an unconventional challenge for the treatment of high-risk tumors. Historically, viruses such as hepatitis, Epstein–Barr, West Nile, and adenovirus have been repurposed to exploit their oncolytic properties (19, 20). The majority of recent oncolytic viruses, such as paramyxoviruses, herpes viruses, and vaccinia viruses, are genetically engineered derivatives designed to attenuate these concerns (21–23). Recently, T-VEC, a modified herpes simplex virus, was approved by the FDA for a subset of patients with melanoma (24).

Zika virus (ZIKV) has emerged as an alternative oncolytic agent. As a member of the *Flaviviridae* family (25), ZIKV has a mild clinical course, with 80% of infections being asymptomatic and the majority of symptomatic infections marked by fever, conjunctivitis, and self-limiting rash in both children and non-pregnant adults (26). The discovery of acute maternal-fetal ZIKV syndrome led investigators to determine that the infection of neuronal progenitor cells was the underlying cause of congenital microcephaly, pointing to these dividing cells as a preferential target of ZIKV infection (27–29). Since then, numerous groups have exploited the oncolytic activity of ZIKV as a potential therapeutic agent for an array of cancers with a central nervous system (CNS) background, including glioblastoma (30–32), embryonal CNS tumors (33), and glioma (34, 35). Extrapolating upon this, our own studies confirmed a profound oncolytic sensitivity of neuroblastoma to ZIKV, dependent on the expression of the membrane protein CD24 (36).

Cluster of differentiation (CD)24 is a glycophosphatidylinositol-linked sialoglycoprotein, which contains highly variable glycosylation that is cell type specific (37). It is typically expressed on progenitor cells and metabolically active cells, including hematopoietic cells such as B cells, T cells, and neutrophils, as well as on non-hematopoietic cells such as neural cells and epithelial stem cells (38). It is also recognized as a cancer stemness marker and is correlated with both tumor progression and poor prognosis in numerous cancers (39, 40). Although the function of CD24 in many cell types is uncertain, it has been identified as a biomarker for neural stem cell differentiation and acts as a cell adhesion molecule crucial for neural development (41, 42). CD24 is commonly expressed in neuroblastomas and is associated with tumor differentiation and the emergence of anaplastic histologic features (43).

This study aimed to characterize the oncolytic effects of ZIKV on neuroblastoma using *in vivo* models, including both high-risk MYCN-amplified and non-amplified tumors. ZIKV application yielded a tumoricidal effect and a significant survival advantage for the host. These results correlate with the expression of CD24 in all neuroblastoma tumors, elucidating both the simultaneous proliferative advantage for tumor expression of CD24 and its consequent alternative role as a predictor of ZIKV permissiveness. This demonstrates that viral sensitivity is CD24 dependent and advances the option for its use as a prognostic marker in an array of human cancers, offering an alternative therapy for both primary and multidrug-resistant recurrent cases.

## Materials and Methods

### Cell Lines, Viruses, and Culture Conditions

SK-N-AS (RRID:CVCL_1700) and IMR-32 (RRID:CVCL_0346) cells were acquired from the ATCC (catalog nos. CRL-2137 and CCL-127, respectively, 2017). SK-N-AS cells were cultured in DMEM + GlutaMAX (catalog no. 10566024, Gibco Life Sciences) supplemented with 10% FBS (catalog no. A4766801, Thermo Fisher Scientific). SK-N-AS/VO (Vector Only) and SK-N-AS/CD24 were cultured as described above, with 6 µg/mL blasticidin (catalog no. J67216.8EQ, Thermo Fisher Scientific) to maintain selection (36). IMR-32 cells were cultured in Minimum Essential Medium (MEM) Alpha + GlutaMAX (catalog no. 32561102, Gibco Life Sciences) supplemented with 10% FBS. SMS-KAN (RRID:CVCL_7131) and SMS-KANR (RRID:CVCL_7132) cells were acquired from the Children's Oncology Group (Columbus, OH, 2017). SMS-KAN and SMS-KANR cells were cultured in HyClone RPMI1640 (catalog no. SH30096LS, GE Healthcare Life Sciences) supplemented with 10% FBS. All of the cell lines used in this study were screened for biological markers originally used to identify these neuroblastoma cells (44). This was performed by examining the qRT-PCR gene expression profiles of CDKN2A, p73, MYCN, and p53 (45–47). The cell lines were thawed and passaged twice prior to validation as well as tested for *Mycoplasma* via the Universal Mycoplasma Detection Kit (catalog no. 30-1012K, ATCC) in May of 2020. Cells were incubated with 5 µg/mL Plasmocin (catalog no. ant-mpp, InvivoGen) in culture and maintained at 37°C in 5% CO_2_.

All ZIKV experiments were performed using Zika viral index strain MR766 acquired from the ATCC (catalog no. VR-1838). ZIKV stock was inoculated into Vero cells (RRID:CVCL_0059, catalog no. CCL-81, ATCC) at a multiplicity of infection (MOI) of 0.1. Following inoculation, the infected cells were incubated for 4 days based on cytopathic effects. The medium was then harvested and clarified by low-speed centrifugation to produce a crude stock. Crude stocks were then titered using a plaque assay and used in any *in vitro* study. ZIKV for *in vivo* work was prepared as described previously, with an added ultracentrifugation step (36). The collected medium and 30% glycerol solution were ultracentrifuged at 24,000 RPM for 3 hours at 15°C. Pellets were resuspended in serum-free DMEM (catalog no. 11965092, Thermo Fisher Scientific) and retitrated by a plaque assay. The stocks were flash frozen in liquid nitrogen and stored at −80°C.

### Tissue Microarray Staining

Neuroblastoma and peripheral nerve tissue microarray (TMA) slides (catalog no. NB642c, Amsbio) containing 32 cases/64 sections were deparaffinized with 100% xylene and rehydrated sequentially in 100%, 95%, and 70% ethanol. The slide was unmasked using heat-induced epitope retrieval for 10 minutes at 98°C in pH 7.8 buffer, and blocked in 2.5% normal goat serum (catalog no. 30023, Vector Labs) in 1x PBS-T. It was then incubated overnight at 4°C in a closed humidified chamber with CD24 (SN3) primary antibody (catalog no. sc-19585, Santa Cruz Biotechnology) diluted 1:20 in blocking solution (catalog no. SP-6000, Vector Labs). The following day, the secondary antibody, goat anti-mouse IgG [LSBio (LifeSpan) catalog no. LS-C30119-50, RRID:AB_905736], was added to the tissue, followed by ImmPACT DAB EqV staining for 45 seconds (catalog no. SK4103, Vector Laboratories). TMA was visualized using a BZ-X800LE Microscope (Keyence Corp) and quantified using the FIJI ImageJ software (ImageJ, RRID:SCR_003070).

### *Ex Vivo* Sample Extraction and Study

A newly resected neuroblastoma tumor from the posterior mediastinum was acquired following Institutional Review Board (IRB) approval (IRB Net # 788229-1). The tumor fragment was acquired within hours of resection, washed in PBS × 2 (DPBS, catalog no. 14040-133, Thermo Fisher Scientific), treated with Accumax (catalog no. 00-466656,Thermo Fisher Scientific) for 5 minutes at 37°C, gently vortexed, and the sheared fragments were collected by centrifugation at low speed (1,200 rpm for 2 minutes). This was performed 12 times to acquire dissociated cells and tissues. Dissociated material (10 mg) was placed into wells of 12-well plates, raised in 1 mL of media [DMEM, Iscove's Modified Dulbecco's Medium (IMDM), MEMα, or RPMI1640], and treated with 0 (uninfected), 1,000, 10,000, or 100,000 pfu ZIKV. IMDM (catalog no. SH30228FS, Thermo Fisher Scientific) was supplemented with 20% FBS and 1X ITS (5 µg/mL insulin, 5 µg/mL transferrin, 5 ng/mL selenium, catalog no. 41400045, Thermo Fisher Scientific). All cultures were incubated with 5 µg/mL Plasmocin (InvivoGen) and maintained at 37°C in 5% CO_2_. Bright-field imaging was performed using an EVOS M5000 (catalog no. AMF5000, Thermo Fisher Scientific) at 40x magnification.

### *Ex Vivo* Sample Cytotoxicity Assays

One milligram of dissociated *ex vivo* neuroblastoma material was placed into the wells of a 96-well plate, raised in 100 µL of media (DMEM, IMDM, MEMα, or RPMI1640), and treated with either 0 (uninfected), 1,000, 10,000, or 100,000 pfu ZIKV. Sextuplicate wells were established for each medium and each Zika treatment concentration. Cultures were then treated with CellTox Green (catalog no. G8741 Promega) in 100 µL of additional medium and measured every 24 hours for 5 days. All cultures were incubated with 5 µg/mL Plasmocin (InvivoGen) and maintained at 37°C in 5% CO_2_. The results were normalized to uninfected (0) controls.

### Animal Studies

All mice were purchased from Taconic Biosciences (Model # NCRNU-F), genotyped before shipment, housed in pathogen-free facilities using the Excluded Flora health standard, and fed on an NIH #31M Rodent Diet. These studies exclusively utilized 5 to 7 weeks old female NCr nude athymic mice (nomenclature: *CrTac:NCr-Foxn1^nu^*), maintained under pathogen-free conditions, and subjected to 12-hour light/dark cycles. The experiments were approved by the Institutional Animal Care and Use Committee (IACUC) of the Burnett School of Biomedical Sciences at the University of Central Florida (Orlando, FL). Experimenters were not blinded to the subjects because tumor-bearing mice were visibly different from controls. We did not check for sample sizes using a power analysis because our study is reporting statistical changes between groups.

### Tumor Studies

All cell lines were injected subcutaneously into the hind flank regions of mice. The injection solutions were a composite of 50% PBS and 50% Matrigel Matrix (catalog no. 354234, Corning) and raised at 5 × 10^7^ cells/mL. The injection volume was 100 µL (containing 5 × 10^6^ cells/injection). The length and width of the tumors were measured and calculated using the formula mm^3^ = ½ (*W*^2^ × *L*); using Mitutoyo ABSOLUTE digimatic calipers, (catalog no. 500-150-30). Tumors were allowed to grow until the average tumor size was approximately 300 mm^3^ (mice with tumors >500 mm^3^ or <100 mm^3^ were excluded from the study). The mice were then randomized to equilibrate absolute tumor sizes for two groups: vehicle and virus. Each group contained a minimum *n* of 6 for the study. The vehicle consisted of PBS, whereas the virus consisted of 2 × 10^6^ ZIKV pfu cells raised in PBS. Injection volumes constituted 100 µL of the total volume delivered via intratumoral administration. The tumors were treated only once per study. At the time of euthanasia, resected tumor samples were frozen in liquid nitrogen and stored at −80°C for further analysis.

### Tumor Survey

Tumors and the health of mice were monitored and measured three times per week for both tumor size and fold change. The study was concluded 30 days after confirmation of no static changes in tumor size in the virus-treated mice. Vehicle-treated mice were monitored for the same period for changes in health and morbidity. Regardless of the group, once a tumor measured >2,000 mm^3^, the mice were retired from the study as criteria for humane euthanasia, and their tumors were resected and stored for further study.

### Tumor Time Course

Tumors and mice were monitored and measured (for tumor size and weight) at precise timepoints, as best determined from their respective tumor surveys (days 0, 2, 5, and 7 for IMR-32 cells; days 0, 4, 7, and 10 for SK-N-AS cells). At each timepoint, 3 mice from each group (vehicle- and virus-treated) were euthanized, and their tumors were harvested along with the kidneys, spleen, liver, heart, and brain of each mouse.

### CD24 Exogenous Expression in SK-N-AS Tumor Study

This study included wild-type (WT) SK-N-AS (ICLC catalog no. HTL95026, RRID:CVCL_1700), SK-N-AS/VO, and SK-N-AS/CD24 cells divided into two groups: vehicle and virus. Each group contained a minimum *n* of 6 for the study. Tumors and mice were monitored and measured periodically for both tumor size and fold change in tumor growth from the point of treatment. The study was concluded as indicated above.

### Tumor Survival Study

The tumors and health of the mice were monitored and periodically measured for tumor size. For survival studies, animal welfare guidelines dictated that once tumors measured > 2,000 mm^3^, mice were euthanized to meet the humane endpoint criteria. The study was concluded once all mice from the vehicle group retired. Their tumors were then resected and stored for further study.

### RNA Isolation and Quantitative PCR

Total cellular RNA was purified from cell and tissue samples using a Direct-zol RNA Miniprep kit (catalog no. R2052, Zymo Research) and purified according to the manufacturer's instructions. The RNA concentration in each sample was determined using a Nanodrop 2000c spectrophotometer (catalog no. ND-2000, Thermo Fisher Scientific). Then, the mRNA was converted into cDNA using the Applied Biosystems High-Capacity cDNA Reverse Transcription Kit (catalog no. 4368814, Thermo Fisher Scientific). qRT-PCR was performed using the CFX384 Touch Real-Time PCR Detection System (Bio-Rad Laboratories, RRID:SCR_008426) with TaqMan Fast Advanced Master Mix (catalog no. 4444556, Applied Biosystems, Thermo Fisher Scientific). TaqMan gene expression assays were used to amplify CD24 and GAPDH (catalog no. 4331182, Assay IDs Hs02379687_s1 and Hs02786624_g1, Applied Biosystems, Thermo Fisher Scientific) in triplicate. The results are reported as the mean ± SEM.

### Absolute Quantification PCR

Gene-specific primers for absolute quantification PCR were designed from their respective gene sequences using PrimerQuest (Integrated DNA Technologies) to generate sequences for PCR amplicons spanning exon-exon junctions. The gene-specific PCR primer oligonucleotide sequences used were as follows: GAPDH, sense primer 5′-ACATCGCTCAGACACCATG-3,′ and anti-sense primer 5′-TGTAGTTGAGGTCAATGAAGGG-3′; Zika ENV, sense primer 5′-CGCAGGATCATAGGTGATGAAG-3,′ and anti-sense primer 5′-CCTGACAACACTAAAATTGGTGC-3′. Further processing was performed as described previously (36).

### IHC of Neuroblastoma Tumors

Fourteen control and nine ZIKV-treated specimens for SK-N-AS tumors and 12 control and eight ZIKV-treated specimens for IMR-32 tumors were fixed in 10% buffered formalin. The specimens were then subjected to gross examination. The SK-N-AS control tumors averaged 9.8 mm in the greatest dimension, ZIKV-treated, 5.0 mm. The IMR-32 control tumors averaged 8.8 mm in the greatest dimension, ZIKV-treated, 6.8 mm. All the tissue samples were processed using a Leica ASP6025 Processor. The resultant formalin-fixed paraffin-embedded tissue was cut on a microtome into 0.5-µm sections, stained with hematoxylin and eosin (H&E), and examined microscopically using an Olympus BX43 microscope at a magnification of 40x with a 10x zoom. In addition, IHC was performed using antibodies against CD24 [Invitrogen, MA5-11828 (SN3)] at a 1:50 dilution and Zika NS1 (GeneTex, catalog no. GTX133307) at a 1:500 dilution, probed with horseradish peroxidase (HRP)-conjugated secondary antibodies (catalog no. 62-6520, Thermo Fisher Scientific), and assayed using a Leica Bond Maxfive. H-scores for NS1 staining were calculated for equal numbers of treated and control animals. H-scoring of IHC assays is an attempt to capture the number of positively reacting cells and the intensity of staining. The staining intensity was graded from 0 to 3, with 0 indicating no staining and 3 indicating maximal staining. A score of 1 or 2 represented light or moderate staining intensity, respectively.

### Construction of IMR-32 Cells Stably Expressing CD24 Short Hairpin RNA

IMR-32 cells were seeded at 2.5 × 10^5^/well in 6-well plates and transfected using Fugene 6 (Promega Corp.) with 2 µg of three separate pCHI- CD24 short hairpin RNA (shRNA) plasmids (Genecopoeia, catalog nos. HSH070733-CH1-a, b, and c as well as a negative control: CSHCTR001-CH1). Transfection was allowed to continue for 6 hours, after which the medium was removed, and the cells were washed with PBS. Fresh medium was added 24 hours after transfection, and the cells were selected at 2 µg/mL with puromycin (catalog no. B7587, Life Technologies Corp.). The selection was continued for 10 days until individual colonies could be isolated.

### Validation of the Stable Knockdown of CD24 in IMR-32 Cells

qRT-PCR was used to measure CD24 mRNA levels in IMR-32/WT, IMR-32/shRNA control (con), and IMR-32/CD24 shRNA cell samples. Samples were prepared as described above. TaqMan gene expression assays were used for CD24, normalized to GAPDH, and amplified in triplicate. The results are reported as the mean ± SEM.

Western blot analysis was also performed, using 2.5 × 10^5^ cells of each selected IMR-32 sample (as above). These cells were then boiled in NuPAGE LDS Sample Buffer (catalog no. NP0007, Thermo Fisher Scientific), and proteins were separated by electrophoresis on 4%–12% NuPAGE Bis-Tris denaturing polyacrylamide gels (catalog no. NP0321BOX, Thermo Fisher Scientific). Proteins were transferred to nitrocellulose membranes (0.2 µm, catalog no.1620112, Bio-Rad,) and probed with the following primary antibodies: anti-CD24 (Thermo Fisher Scientific, catalog n. MA5-11828, RRID:AB_10983158) at 1/200 and anti-GAPDH (catalog no. FL-335: sc-25778, Santa Cruz Biotechnology) at 1/2,000. Blots were probed with HRP-conjugated secondary antibodies (Invitrogen, catalog no. 62-6520, Goat anti-Rb, catalog no. 65-6120) and visualized using enhanced chemiluminescence chemiluminescence (catalog no. 32106, Pierce, Thermo Fisher Scientific).

### Bright-field Analysis of CD24 Stable Knockdown in IMR-32 Cells Treated with ZIKV

A total of 2.5 × 10^5^ cells per well were placed into 6-well plates with each selected IMR-32 (CLS catalog no. 300148/ p666_IMR-32, RRID:CVCL_0346) sample (including WT IMR-32, IMR-32/shRNA con, and IMR-32/CD24 shRNA), raised in 2 mL of MEMα medium, and treated with either 0 (No Zika), 1, or 10 MOI of ZIKV. All cultures were incubated with 5 µg/mL Plasmocin (InvivoGen) and maintained at 37°C in 5% CO_2_. Bright-field imaging was performed using an EVOS M5000 (Invitrogen, Thermo Fisher Scientific) at 40x magnification with a 4x zoom.

### Cytotoxicity Assays for CD24 Stable Knockdown in IMR-32 Cells Treated with ZIKV

Cells (2 × 10^3^) of each selected IMR-32 sample (including WT IMR-32, IMR-32/shRNA con, and IMR-32/CD24 shRNA) were placed into wells of a 96-well plate, raised in 100 µL of MEMα medium, and treated with either 0 (No ZIKV) or an MOI of 10 of ZIKV. Sextuplicate wells were established for each cell line and treatment concentration. Cultures were then treated with CellTox Green (Promega) in 100 µL of MEMα medium and measured every 24 hours for 5 days. All cultures were incubated with 5 µg/mL Plasmocin (InvivoGen) and maintained at 37°C in 5% CO_2_. The results were normalized to those of the uninfected (No ZIKV) controls.

### Statistical Analysis

All statistical analyses within this article were performed on a minimum of triplicate experimental samples. The data were then analyzed and combined in GraphPad Prism 7 (GraphPad Prism, RRID:SCR_002798) and assessed for a *P* value < 0.05, analyzed by one-way ANOVA. An unpaired *t* test was used for pairwise comparison.

### Study Approval

#### Animal Studies

All animal handling and procedures were approved by the IACUC of the Burnett School of Biomedical Sciences at the University of Central Florida (Orlando, FL; protocol PROTO201900014).

### Data Availability Statement

The data generated in this study are available upon request from the corresponding author.

## Results

### CD24 is Commonly Expressed in Human Neuroblastoma Tumor Isolates and an *Ex Vivo* Isolate is Permissive to Zika Viral Killing

**FIGURE 1:**
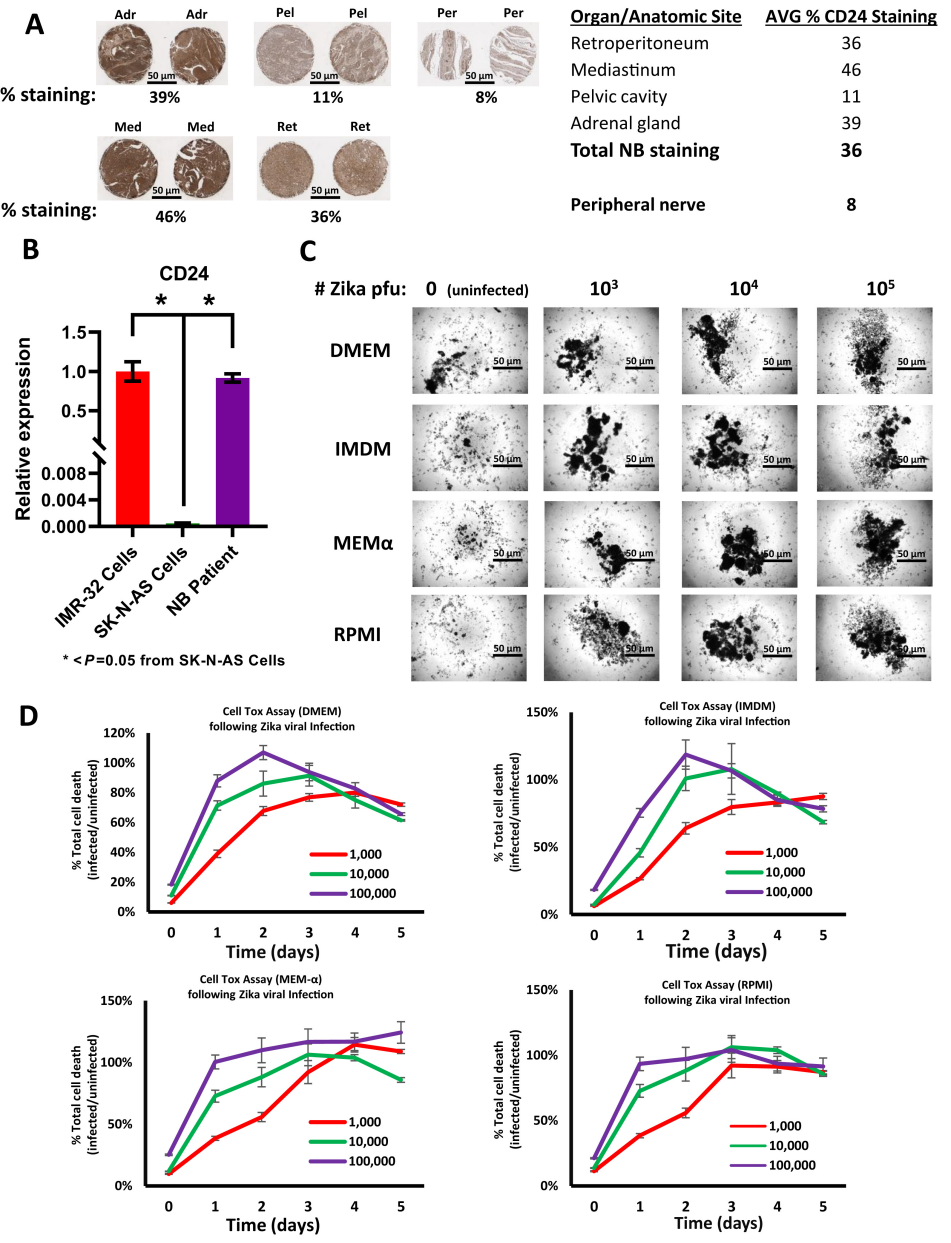
Assessment of neuroblastoma patient samples for CD24 expression and permissiveness of ZIKV in *Ex Vivo* patient isolate. **A,** Human neuroblastoma patient tissue samples stained for expression of CD24. Representative samples include tumors isolated from the Adrenal gland (Adr), Mediastinum/left posterior (Med), Pelvic cavity (Pel), and Retroperitoneum (Ret) as well as control Peripheral nerve (Per) tissue. Visualization of the tissues was performed using Keyence and quantified using FIJI ImageJ. **B,** qRT-PCR was performed comparing IMR-32 cells (*in vitro*), SK-N-AS cells (*in vitro*), and a neuroblastoma (NB) patient tumor isolate (*ex vivo*) for CD24 expression. Expression was normalized to GAPDH. All qPCR data shown are the composite of triplicate wells acquired from triplicate experiments. Error bars represent SD. *, *P* < 0.05 from SK-N-AS Cells, one-way ANOVA for IMR-32 and NB patient. **C,** Bright-field comparison of *ex vivo* neuroblastoma patient sample with and without application of ZIKV. *Ex vivo* tumor was cultured in DMEM, IMDM, MEMα, or RPMI1640 and treated with either 0, 10^3^, 10^4^, or 10^5^ pfu ZIKV. Cultures were assessed over the course of 5 days posttreatment in 40x magnification. **D,** Time course of cellular toxicity in *ex vivo* neuroblastoma patient sample with and without application of virus. Cultures were treated with CellTox Green as well as 0, 10^3^, 10^4^, or 10^5^ pfu ZIKV and measured every 24 hours for 5 days. The results were normalized to uninfected (0) controls. Data shown are the composite of sextuplicate wells, performed in triplicate from independent measurements. Error bars represent SD.

This laboratory's prior experimentation indicated that CD24 is necessary for the permissiveness of ZIKV *in vitro* using neuroblastoma cell lines (36). To substantiate the translational value of this observation, it was necessary to validate CD24 expression in human neuroblastoma tumor isolates. A neuroblastoma tissue array containing 27 cases of neuroblastoma (including primary isolates resected from the region of the adrenal glands, pelvic cavity, mediastinum, and retroperitoneum) was stained for CD24 expression and compared with mature peripheral nerve tissue (Fig. 1A; Supplementary Fig. S1). The results confirmed that the expression of CD24 was significantly higher in neuroblastoma tumors isolated from the adrenal glands, mediastinum, and retroperitoneum, with an average staining of approximately 450% greater than that seen in the peripheral nerve. These data corroborate that CD24 remains well expressed within a broad array of neuroblastoma patient isolates, validating prior observations in neuroblastoma cells.

Similarly, to further examine the potential translational relevance of ZIKV as a tumor therapy, we screened the capacity of Zika viral strain MR766 to induce cytotoxicity in a neuroblastoma *ex vivo* tumor isolate. The tumor was acquired >2 hours after resection from the patient's posterior mediastinum, washed in PBS, and gently sheered enzymatically. A portion of the sample was used to isolate total RNA for qRT-PCR and screen for CD24 expression (Fig. 1B). The results indicated robust expression of CD24 in the neuroblastoma patient sample, comparable to the levels seen in previously sensitive IMR-32 cells, predicting sensitivity to viral killing (as opposed to SK-N-AS cells, whose low expression of CD24 indicated resistance to viral killing). The remainder of the patient sample was placed in an array of media commonly used for culturing neuroblastoma cells. ZIKV was then introduced at dosages of 0 (uninfected), 10^3^, 10^4^, or 10^5^ pfu per medium type and examined every day for 5 days (Fig. 1C). Bright-field imaging confirmed significant clustering of cellular and tissue debris by day 5 in all ZIKV-treated wells, regardless of the dosage or culture medium. In comparison, little debris was detected in the untreated (0 pfu) wells, suggesting that the medium did not contribute to significant differences in cell death, and even the lowest viral dosage (10^3^ pfu) was sufficient to do so. To quantify this process, a cytotoxicity assay was performed on the *ex vivo* sample under the same conditions (Fig. 1D). Our results confirmed a significant increase in cell death for every ZIKV-treated sample on every day after day 0. This increase maximized by day 3 for the highest dosage (10^5^ pfu) and by day 5 for the lowest dosage (10^3^ pfu), regardless of the culture medium. These data validate that ZIKV treatment of a resected neuroblastoma can induce rapid cell death in the tumor in a dose- and time-dependent manner.

### *In Vivo* Modeling of Human Neuroblastoma Tumors Confirms Permissivity of Zika Viral Therapy

**FIGURE 2:**
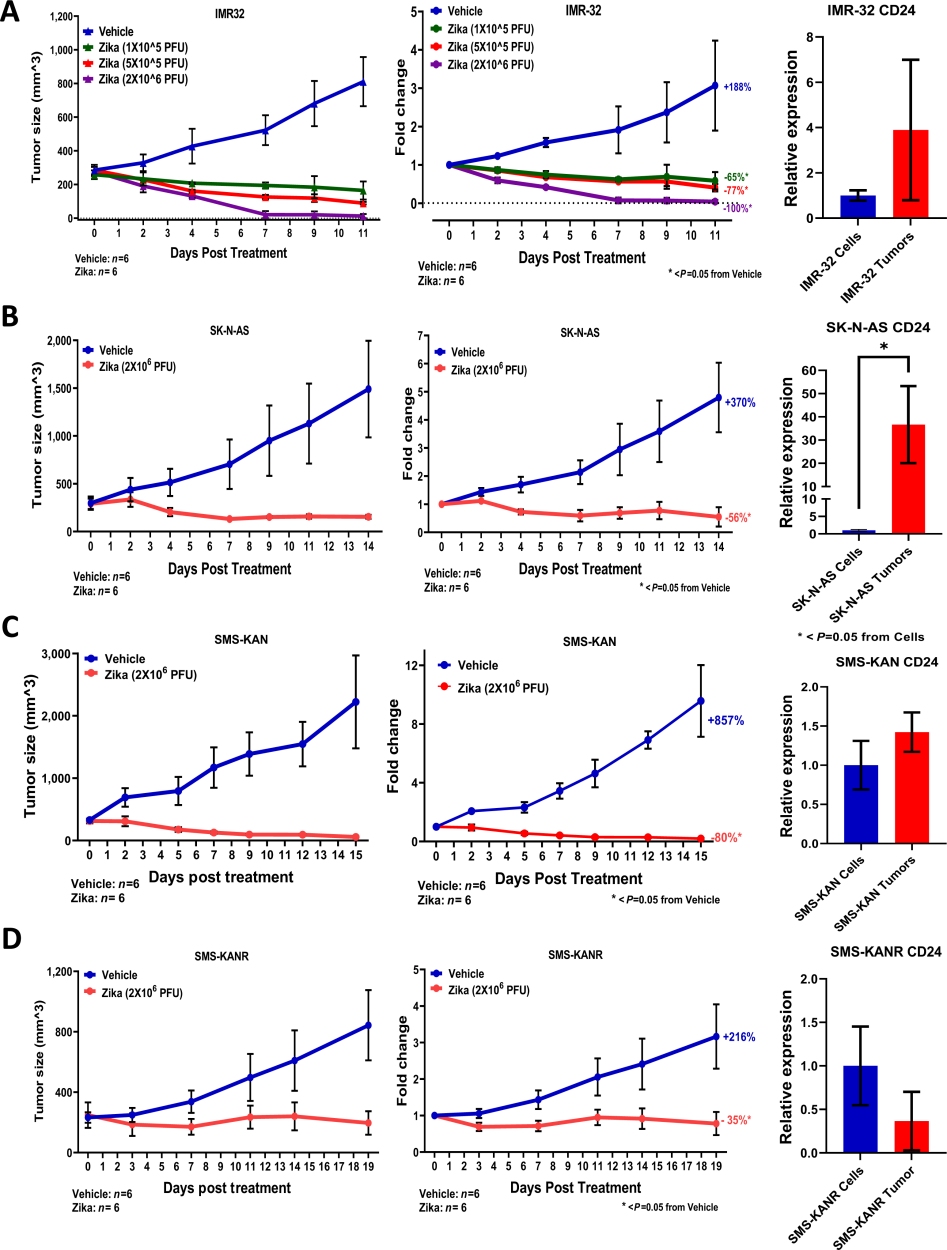
*In vivo* mouse modeling of the Zika viral treatment of neuroblastoma tumors. **A,** Assessment of the dose-dependent application of ZIKV to IMR-32 tumors in NCr nude mice. Data are depicted through day 11 posttreatment. Error bars represent SD. *, *P* < 0.05 from Vehicle, one-way ANOVA for Zika (1 × 10^5^), Zika (5 × 10^5^), and Zika(2 × 10^6^) day 11. **B,** Assessment of ZIKV application to SK-N-AS tumors. Data are depicted through day 14 posttreatment. Error bars represent SD. *, *P* < 0.05 from Vehicle, unpaired *t* test. **C,** Assessment of ZIKV application to SMS-KAN tumors. Data are depicted through day 15 posttreatment. Error bars represent SD. *, *P* < 0.05 from Vehicle, unpaired *t* test. **D,** Assessment of ZIKV application to SMS-KANR tumors. Data are depicted through day 19 posttreatment. Error bars represent SD. *, *P* < 0.05 from vehicle, unpaired *t* test. All studies utilized an *n* = 6 for both vehicle- and Zika-treated cohorts. Virus was introduced once at concentrations of 1 × 10^5^, 5 × 10^5^, or 2 × 10^6^ pfu (compared with vehicle PBS only) in IMR-32 tumors and introduced once at a concentration of 2 × 10^6^ pfu for all other neuroblastoma models. All tumors were assessed for both “tumor size” (mm^3^) and “fold change” in tumor mass (comparing viral-treated tumors and vehicle-treated control tumors normalized to masses established at day 0 posttreatment). Relative expression for CD24 was assessed using qRT-PCR by comparing cells (*in vitro*, prior to introduction into the mouse host) with tumors (*in vivo*, averaged from posttreatment vehicle-treated control tumors) for each neuroblastoma. Expression was normalized to GAPDH. All qPCR data shown are the composite of triplicate wells acquired from triplicate experiments. Error bars represent SD. *, *P* < 0.05 from SK-N-AS Cells, unpaired *t* test.

Our previous study illustrated the permissiveness of ZIKV across a panel of human neuroblastoma cells *in vitro* (36). However, to expand on the *in vitro* data, it is necessary to validate our experiments *in vivo*. We established a human xenograft neuroblastoma tumor model by injecting MYCN-amplified IMR-32 cells into NCr nude mice. A total of 5 × 10^6^ cells were injected subcutaneously into the hind flank regions of mice and tumors were allowed to form. Once the masses averaged approximately 300 mm^3^, the mice were randomized into two groups: vehicle and virus. Injections were then delivered in a total volume of 100 µL via intratumoral administration. ZIKV was then injected at the tumor margin to allow the virus to directly access the tumor cells. This allowed the use of a lower viral dose as opposed to injecting at a distant location, which could dilute the viral dose prior to reaching the target cells. Tumors were treated with either 1 × 10^5^, 5 × 10^5^, or 2 × 10^6^ pfu of ZIKV (compared with PBS vehicle controls). Tumor size and fold change in tumor mass were measured over a 4-week period (Fig. 2A). The results indicated that all doses of ZIKV led to a net loss in tumor size and mass, although a dosage of 2 × 10^6^ pfu led to a complete loss of tumor mass by day 7. These tumors showed no recovery of tumor mass even after a 4-week posttreatment period, leading us to use this dosage for the remainder of our studies. In addition, no mice developed any symptoms of ZIKV infection, including conjunctivitis, fever, or skin rashes. Given the importance of CD24 in viral permissivity *in vitro*, we compared the expression of CD24 in IMR-32 cells (*in vitro*) to that in IMR-32 tumors (*in vivo*) by qRT-PCR. These results revealed that the tumors retained robust expression of CD24, with some variation in expression, although this difference was not statistically significant when compared with *in vitro* cells.

In our previous study, we found that non–MYCN-amplified SK-N-AS neuroblastoma cells were significantly resistant to ZIKV permissivity and viral killing *in vitro*. However, *in vivo* modeling of SK-N-AS tumors revealed surprising results. Treatment of these tumors with ZIKV led to a loss of the tumor mass by day 4 and an eventual loss of >50% of the initial tumor mass by day 14 (Fig. 2B). Likewise, these tumors showed no recovery of the tumor mass through week 4 posttreatment and no evidence of viral symptoms. Given that resistance to Zika permissivity *in vitro* was shown to be dependent on the low expression of CD24, which could be overcome by its ectopic expression*,* we compared the levels of CD24 in SK-N-AS cells (*in vitro*) with those in SK-N-AS tumors (*in vivo*) by qRT-PCR. The results revealed that CD24 expression increased approximately 20-fold in SK-N-AS tumors compared with that in cells, perhaps explaining the acquired sensitivity to viral susceptibility *in vivo*.

Although IMR-32 and SK-N-AS cells represent a primary tumor isolate and a recurrent tumor, respectively, they represent separate neuroblastoma tumors in unrelated patients. To further address the extent of Zika permissivity, we performed *in vivo* modeling using a matching patient pair of SMS-KAN cells (a pretreatment primary isolate) and SMS-KANR cells (a posttreatment recurrent isolate). ZIKV treatment of both SMS-KAN and SMS-KANR tumors led to significant suppression of tumor growth, although the SMS-KAN tumors responded more dramatically (a loss of >80% by day 15 compared with only ∼35% loss of the initial tumor mass by day 19 for SMS-KANR; Fig. 2C and D). Consistent with our previous models, neither tumor showed any recovery of mass through week 4 posttreatment, suggesting that ZIKV permissivity could suppress tumor growth regardless of statis as a primary versus recurrent tumor isolate. Similar to IMR-32, the expression of CD24 did not reveal any significant variation between the *in vitro* (cells) and *in vivo* (tumor) samples. A comparison of CD24 expression between all the *in vitro* and *in vivo* samples confirmed that only the SK-N-AS samples demonstrated significant differential expression and were, overall, dramatically lower expressed in their *in vitro* samples (Supplementary Fig. S2).

### A Time Course of Zika Viral–treated Neuroblastoma *In Vivo* Models Indicates a Rapid Loss of tumor Mass with a Lack of Viral Shedding

**FIGURE 3:**
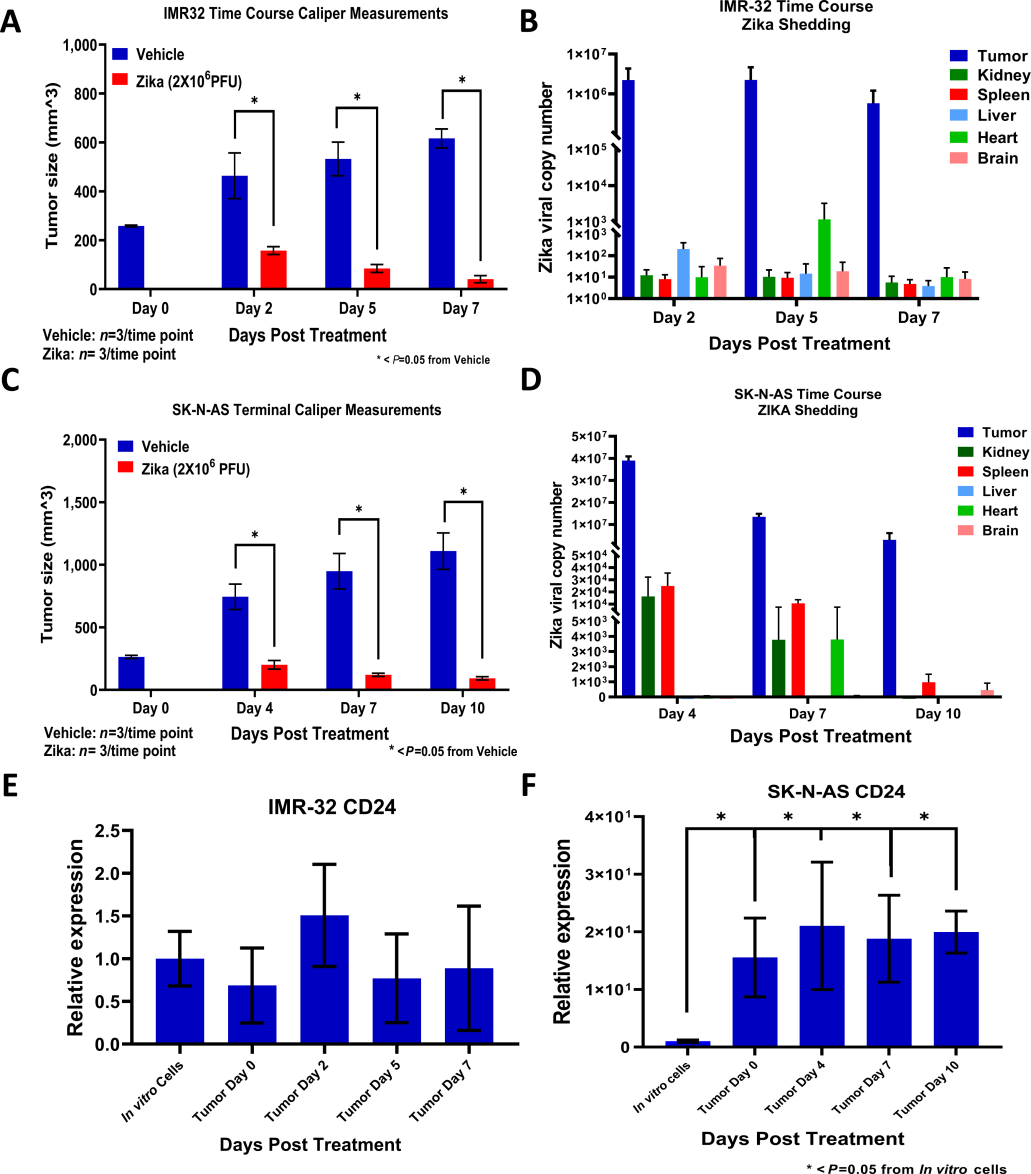
Time course of the *In vivo* modeling of the Zika viral treatment of neuroblastoma tumors. ZIKV was introduced once at a concentration of 2 × 10^6^ pfu for all tumors in NCr nude mice. **A,** Time course assessment of the application of ZIKV to IMR-32 tumors. Tumor size was measured at day 0, 2, 5, and 7 posttreatment compared with vehicle control treatment. Each timepoint within the study utilized an *n* = 3 for both vehicle- and Zika-treated cohorts. Error bars represent SD. *, *P* < 0.05 from Vehicle, unpaired *t* test, days 2, 5, and 7. **B,** Viral copy number was also measured in vehicle control treated mice for each timepoint in the IMR-32 tumor model, measuring the tumor, kidneys, spleen, liver, heart, and brain by absolute quantification PCR. **C,** Time course assessment of the application of ZIKV to SK-N-AS tumors. Tumor size was measured at day 0, 4, 7, and 10 posttreatment compared with vehicle control treatment. Each timepoint within the study utilized an *n* = 3 for both vehicle- and Zika-treated cohorts. Error bars represent SD. *, *P* < 0.05 from vehicle, unpaired *t* test, days 4, 7, and 10. **D,** Viral copy number was also measured in vehicle control treated mice for each timepoint in SK-N-AS tumor models, measuring the tumor, kidneys, spleen, liver, heart, and brain by absolute quantification PCR. **E,** Relative expression for CD24 was assessed using qRT-PCR at each timepoint for IMR-32 tumors. **F,** Relative expression for CD24 was assessed using qRT-PCR at each timepoint for SK-N-AS tumors. Both qRT-PCR studies compare cells with tumors for each neuroblastoma. Expression was normalized to GAPDH. All qPCR data shown are the composite of triplicate wells acquired from triplicate experiments. Error bars represent SD. *, *P* < 0.05 from *In vitro* cells, one-way ANOVA for tumor day 0, 4, 7, and10.

To further examine the impact of ZIKV treatment on neuroblastomas, we performed an *in vivo* time course analysis following the application of the virus to the tumors. Given that a nearly total loss of tumor mass was reached by day 7 posttreatment for IMR-32 tumors, we measured and acquired tumors on days 0, 2, 5, and 7 posttreatment (Fig. 3A; Supplementary Fig. S3A). The results confirmed that ZIKV-treated IMR-32 tumors lost size and mass by day 2, yielding less than half of that seen in vehicle-treated tumors, which continued to grow over that period, averaging approximately 157 mm^3^ for treated tumors compared with approximately 463 mm^3^ for vehicle. Likewise, on days 5 and 7, additional losses in size yielded tumors that retained only a fraction of the control tumor size, with ZIKV-treated tumors continuing to decrease in growth, averaging approximately 84 mm^3^ and approximately 40 mm^3^ compared with approximately 532 mm^3^ and approximately 616 mm^3^ for the vehicle-treated tumors, respectively. To assess viral shedding in the host, an absolute quantification of ZIKV copy number was performed in the tumors, kidneys, spleen, liver, heart, and brain of the host (Fig. 3B). The results indicated that although viral content reached approximately 2.2 × 10^6^ copies within the tumor, only approximately 10^2^ copies could be measured in any organ by day 2, and titers never breached greater than approximately 10^3^ copies at any timepoint in any organ. These values diminished to less than 10^1^ copies by day 7 in all organs, indicating that viral shedding was not permissive in the host.

Similarly, an *in vivo* time course was performed in SK-N-AS tumors, revealing that the maximal loss of tumor mass was reached by day 10 posttreatment. Thus, measurements and acquisition of tumors were performed at days 0, 4, 7, and 10 posttreatment (Fig. 3C; Supplementary Fig. S3B). These results were consistent with those seen in ZIKV-treated IMR-32 tumors, with significant losses in both size and mass by the first timepoint (day 4), yielding less than a third of the results seen in vehicle-treated tumors (averaging ∼200 mm^3^ for treated tumors compared with an average of ∼744 mm^3^ for vehicle). Likewise, days 7 and 10 yielded additional losses in the size and mass of the tumors compared with vehicle-treated tumors (averaging ∼120 mm^3^ and 92 mm^3^ treated compared with 948 mm^3^ and 1,109 mm^3^ vehicle, respectively). Further assessments of these timepoints for evidence of viral shedding revealed copies on the order of 10^4^ in both the kidneys and spleen on day 4 (compared with >3 × 10^7^ copies in the tumor itself; Fig. 3D). However, these values diminished by day 7 and dropped to <10^3^ copies by day 10 (compared with 3 × 10^6^ copies remaining in the tumor). Together, these data suggest that greater ZIKV production from the tumor may contribute to greater viral shedding in the host organs. Ultimately, this did not influence the permissiveness of the host, which did not support viral production, as no mice developed symptoms and all titers decreased with time.

Both sets of tumors acquired from their respective time courses were also screened for the expression of CD24 and compared with the *in vitro* cells used to establish these tumors (Fig. 3E and F). These results validated those seen previously, with IMR-32 tumors showing some variation in CD24 expression over time, but the difference was not statistically significant. Likewise, the SK-N-AS tumors indicated approximately 15- to 20-fold increases in CD24 expression compared with their *in vitro* cells, which were maintained throughout the time course.

### The Presence of Zika Viral Protein NS1 Correlates with Cell Death in Zika-treated Neuroblastoma Tumors

**FIGURE 4:**
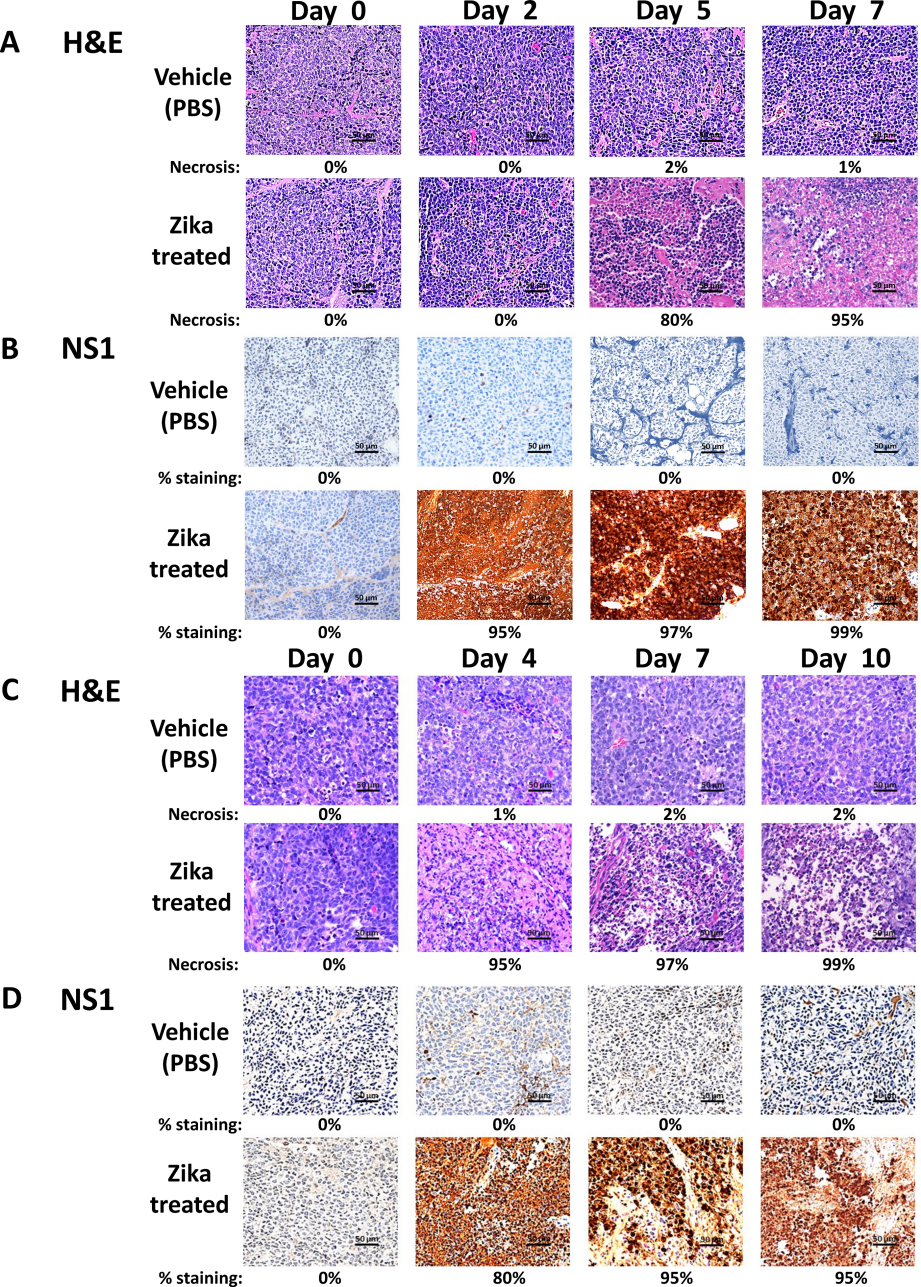
Evaluation of the Zika viral time course on neuroblastoma tumors by IHC. ZIKV-treated tumors were compared with vehicle-treated tumors at each timepoint. **A,** IMR-32 tumors were H&E stained at day 0, 2, 5, and 7 posttreatment. Percent necrosis is indicated under each image. **B,** IMR-32 tumors were stained using a Zika viral NS1 protein antibody at day 0, 2, 5, and 7 posttreatment. **C,** SK-N-AS tumors were H&E stained at day 0, 4, 7, and 10 posttreatment. Percent necrosis is indicated under each image. **D,** SK-N-AS tumors were stained using a Zika viral NS1 protein antibody at day 0, 4, 7, and 10 posttreatment. Tissues were stained using a Leica Bond Maxfive and images were generated using an Olympus BX43 at 40x magnification with a 10x zoom.

To connect the loss of the neuroblastoma tumor mass with ZIKV production, it is necessary to establish that the presence of ZIKV-infected tissue is associated with cell death within the tumor. Therefore, tumors produced during the time course of ZIKV treatment were resected at each individual timepoint and stained by IHC. The results of the IMR-32 study indicated that tumors treated with ZIKV showed marked necrosis of the tumor cells by its first timepoint (day 2; Fig. 4A), whereas uninfected tumors showed little to no cell death even at the last timepoint (day 7). By day 5, ZIKV-treated tumors revealed destruction of over 80% of the tumor, yielding almost all necrotic tissue by day 7. Staining of Zika viral protein NS1 was performed to detect the presence and relative abundance of viral production within the tissue (Fig. 4B). NS1 (or non-structural 1) is a Zika viral–specific protein involved in both viral genome replication and virion production (48). Although there is evidence it can be secreted as a glycoprotein, the majority remains intracellular. Most importantly, NS1 is not present in the infecting virion. As a consequence, it is only present once a permissive infection has occurred, and it is commonly used to validate this condition.

The screening for NS1 confirmed it was not expressed in the control tumors but was strongly expressed in the treated tumors. Those treated with ZIKV and harvested on day 2 showed little treatment effect but were strongly positive for NS1, whereas NS1 staining correlated with cell death by days 5 and 7. Staining of human CD24 was performed to validate previous gene expression results observed over the time course. The results revealed robust expression of CD24 within the tumors, regardless of treatment, although detection waned by day 7 in treated tumors, possibly because of cellular necrosis (Supplementary Fig. S4A).

Consequently, the SK-N-AS time course revealed similar results, with marked necrosis in treated tumors by day 4 progressing to the destruction of over 95% of the tumor by day 10 (Fig. 4C). The untreated tumors showed no significant necrosis at any timepoint. The staining of NS1 indicated that it was strongly positive in the treated tumors by day 4 and remained so throughout the time course (Fig. 4D). Only limited background staining could be detected in the vehicle-treated tumors. Likewise, CD24 was detected in SK-N-AS tumors (Supplementary Fig. S4B), and its expression was also rather stable, only diminishing in treated tumors by day 10.

### The Exogenous Expression of CD24 in SK-N-AS Tumors Positivity Regulates Tumor Growth While Simultaneously Correlating with Increased Loss of Tumor Mass Upon Treatment with ZIKV

**FIGURE 5:**
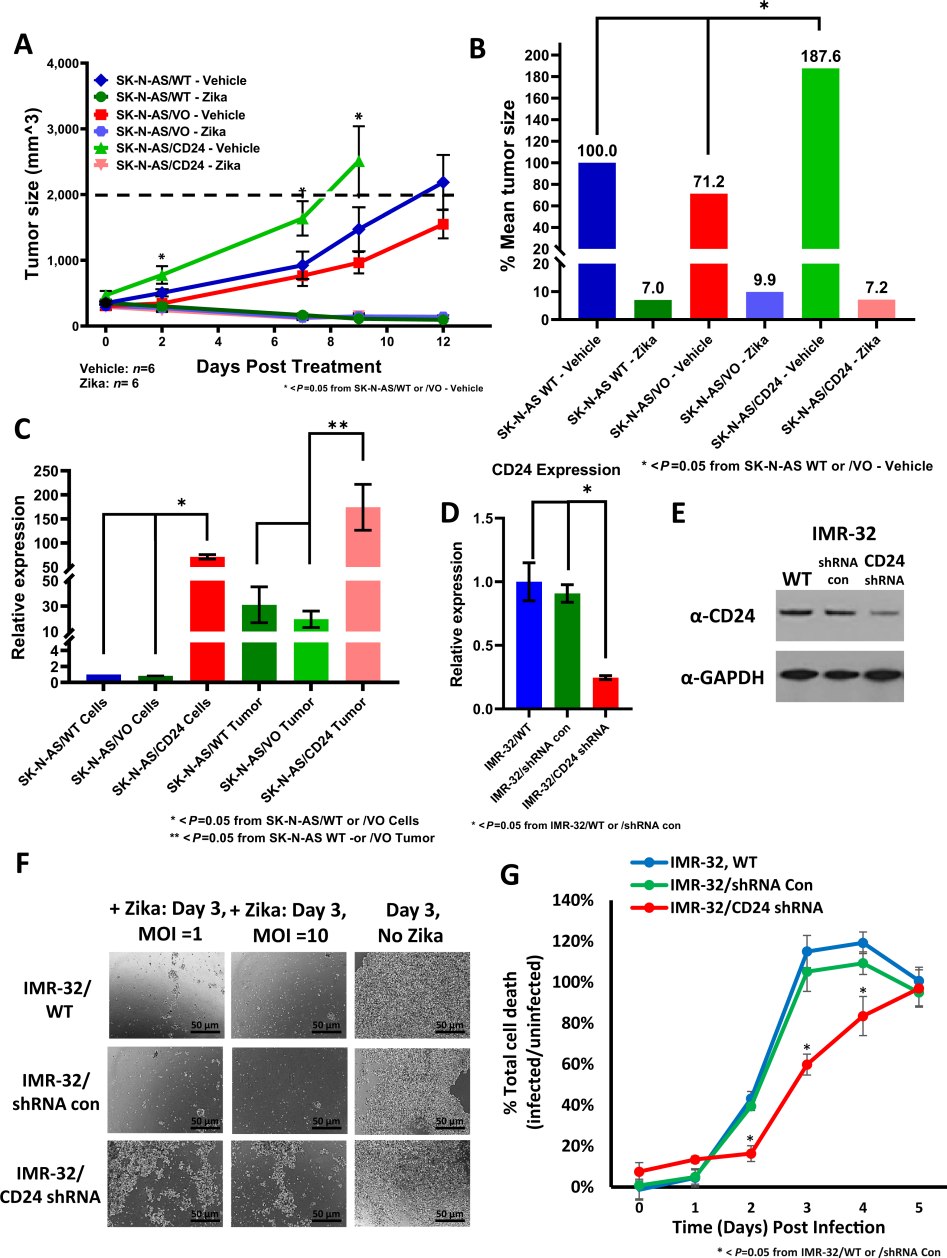
Effect of Zika viral treatment on CD24-exogenously expressing SK-N-AS tumors and CD24 stably knocked-down IMR-32 cells. ZIKV was introduced once at a concentration of 2 × 10^6^ pfu for all tumors. **A,** Evaluation of the application of ZIKV to SK-N-AS/WT, SK-N-AS/VO (Vector Only control), and SK-N-AS/CD24 (stable exogenous expression of CD24) tumors in NCr nude mice, measuring for changes in tumor size (mm^3^). Data are depicted through day 12 posttreatment. All comparisons utilized an *n* = 6 for both vehicle- and Zika-treated cohorts. Error bars represent SD. *, *P* < 0.05 from SK-N-AS/WT or /VO—vehicle, one-way ANOVA for days 2, 7, and 9. **B,** Comparison of the mean tumor size between viral-treated versus vehicle-treated tumors for SK-N-AS/WT, SK-N-AS/VO, and SK-N-AS/CD24, measured at day 9 posttreatment. Tumor sizes were normalized to vehicle-treated SK-N-AS/WT. Error bars represent SD. *, *P* < 0.05 from SK-N-AS/WT or /VO—vehicle, one-way ANOVA. **C,** Relative expression for CD24 was assessed using qRT-PCR comparing cells (*in vitro*) with tumors (*in vivo*) for SK-N-AS/WT, SK-N-AS/VO, and SK-N-AS/CD24. Error bars represent SD. *, *P* < 0.05 from SK-N-AS/WT or /VO—cells; **, *P* > 0.05 from SK-N-AS/WT or /VO—tumor, one-way ANOVA. **D,** Relative expression for CD24 was assessed using qRT-PCR comparing IMR-32/WT cells, IMR-32/shRNA con, and IMR-32/CD24 shRNA (stable). All qPCR expression data shown were normalized to GAPDH and are the composite of triplicate wells acquired from triplicate experiments. *, *P* < 0.05 from IMR-32/WT or /shRNA con, one-way ANOVA. **E,** Western blot analysis of CD24 expression in the total cell lysates of IMR-32 cells and stable derivatives. GAPDH was used as a load control. **F,** Bright-field comparison of IMR-32/WT, IMR-32/shRNA con, and IMR-32/CD24 shRNA stable cells on day 3 posttreatment with ZIKV. Cells were treated with either no virus, an MOI = 1, or an MOI = 10 of virus. Images were generated at a 40x magnification with a 10x zoom. **G,** Time course of cellular toxicity in IMR-32/WT, IMR-32/shRNA con, and IMR-32/CD24 shRNA stable cells. Cells were treated with either no virus or an MOI = 10 of virus. Cultures were treated with CellTox Green and measured every 24 hours for 5 days postinfection. The results were normalized to uninfected controls. Data shown are the composite of sextuplicate wells, performed in triplicate from independent measurements. Error bars represent SD. *, *P* < 0.05 from IMR-32/WT or /shRNA con, one-way ANOVA for days 2, 3, and 4.

Our previous reports indicated that the exogenous expression of CD24 *in vitro* increased the permissiveness of SK-N-AS neuroblastoma cells to ZIKV killing (36, 49). This was performed by cloning the cDNA of CD24 from the permissive IMR-32 cells into eukaryotic expression vectors and introducing these vectors stably into SK-N-AS cells. Following validation of exogenous CD24 expression, these cells were then treated with ZIKV along with controls. To corroborate this *in vivo,* we compared SK-N-AS/CD24 stable cells with VO and WT control cells (as previously performed *in vitro*; Fig. 5A; Supplementary Fig. S5). This study revealed that whereas the rate of tumor growth for the vehicle-treated VO and WT tumors was not significantly different, the vehicle-treated SK-N-AS/CD24 tumors grew at a considerably faster pace. This increased rate of growth yielded tumor sizes and fold changes in mass that were significantly larger by day 2 in the controls and remained so throughout the study. By day 9, all vehicle-treated SK-N-AS/CD24 tumors had breached a tumor size of 2,000 mm^3^ and were unenrolled, whereas none of the vehicle-treated VO or WT tumors had done so. It is important to note that no significant differences were detected in the ZIKV-treated tumors, which revealed a lack of tumor growth by day 2 following ZIKV treatment and continued to decrease in tumor size and fold change of mass throughout the study. An analysis of the mean tumor size revealed that the vehicle-treated SK-N-AS/CD24 tumors averaged >80% increased size compared with WT or VO by day 9 (Fig. 5B). Surprisingly, this dramatic enhancement in the rate of tumor growth offered no advantage to SK-N-AS/CD24 ZIKV-treated tumors in their resistance to viral killing. In fact, the remaining tumor sizes following treatment were not significantly different by day 9 in any category, yielding tumor sizes <10% of those measured prior to treatment.

Analysis of CD24 expression in *in vitro* cells by qRT-PCR confirmed that SK-N-AS/CD24 stable cells yielded a 50-fold increase in expression compared with either VO or WT cells (Fig. 5C; Supplementary Fig. S6A and S6B). Although this is a robust increase, it is still less than half the expression observed in WT IMR-32, SMS-KAN, or SMS-KANR cells (Supplementary Fig. S2; ref. 36). Consequently, the *in vivo* VO tumors revealed an expression increase of approximately 20- to 30-fold in CD24 compared with their *in vitro* counterparts, comparable to the expression change seen previously between WT cells and tumors (Figs. 2B and 3F; Supplementary Figs. S2 and S6A). However, what was most surprising was the result of SK-N-AS/CD24 *in vivo* expression. These tumors appeared to further upregulate the expression of CD24 by nearly 300% of that observed in the SK-N-AS/CD24 cells *in vitro* (Fig. 5C; Supplementary Fig. S6C), suggesting that the native mechanisms that endogenously upregulated the WT and VO tumors were also capable of doing so in the CD24-exogenously expressing tumors. Furthermore, it is important to point out that this additional increase in expression correlated with an enhanced tumor growth rate, implying that a supplementary native increase in CD24 expression in exogenously expressing stable cells offered a dose-dependent selective advantage *in vivo*.

### The Stable Knockdown of CD24 Expression in IMR-32 Cells Negativity Regulates Tumorigenicity While Simultaneously Correlating with Increased Resistance to Cell Death Upon Treatment with ZIKV

Given that we examined the effect of exogenous addition of CD24 to a low-expressing neuroblastoma cell line (SK-N-AS), we also examined the impact of the loss of CD24 in a highly expressing neuroblastoma cell line. We generated a stable CD24 shRNA variant in IMR-32 cells and validated the knockdown by both qRT-PCR and Western blotting compared with a stable shRNA control variant and WT cells (Fig. 5D and E). Our data confirmed a >70% loss of mRNA and a significant loss of CD24 protein. We then generated an *in vivo* model using the IMR-32/CD24 shRNA stable cell line. However, despite consistent tumor formation detected in control cells in the murine host, no tumors could be established from the CD24 knockdown variant. Further adjustments were made with alternate shRNAs, injections of increased cellular load, and allowing for more than twice the time offered to WT and control cells for tumor formation. These conditions yielded no success, implying that the loss of CD24 from any of the shRNAs was likely responsible for the failure of the cells to remain tumorigenic in the murine host.

Consequently, we used *in vitro* modeling to assess the permissivity of this variant in response to ZIKV treatment. IMR-32/CD24 shRNA cells, shRNA control cells, and WT cells were infected with ZIKV at MOI of 0, 1, or 10 and examined over the course of 5 days (Fig. 5F). Bright-field imaging revealed that both WT and shRNA control cells yielded approximately 90% cell death by day 3 at an MOI of 1 and >95% cell death at an MOI of 10. By day 3, the CD24 shRNA stable cells showed only approximately 40% cell death at an MOI of 1 and approximately 60% cell death at an MOI of 10, indicating significant resistance to Zika viral killing. A quantitative assessment of cell death by a cytotoxicity assay confirmed a statistically significant suppression in the rate of cell death in the CD24 knockdown variant on days 2, 3, and 4, delaying nearly total cell death from day 3 to day 5, confirming that the stable loss of CD24 can confer resistance to ZIKV killing (Fig. 5G).

### *In Vivo* Modeling of Human Neuroblastoma Tumors Confirms a Dramatic Survival Advantage for Zika Viral Therapy

**FIGURE 6:**
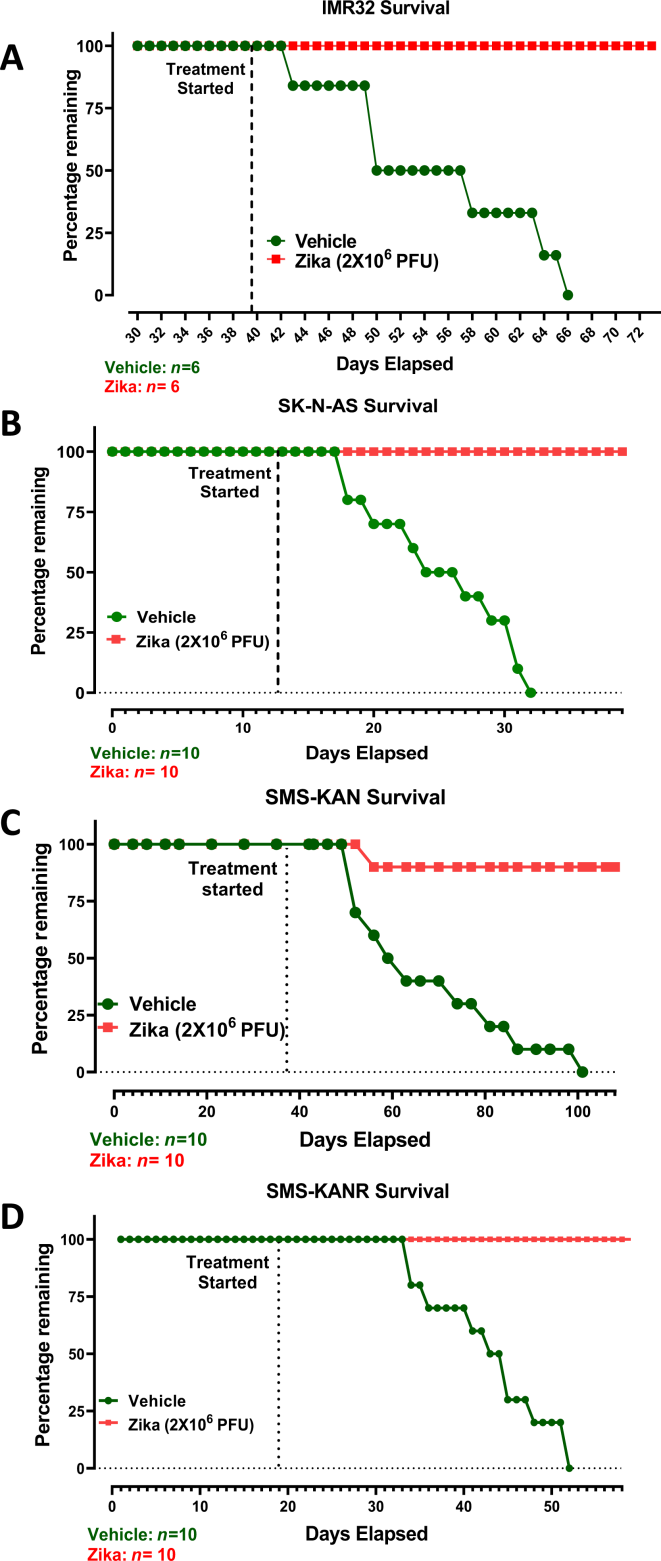
Kaplan–Meier survival curves of the Zika viral treatment of neuroblastoma tumors. ZIKV was introduced once at a concentration of 2 × 10^6^ pfu (compared with vehicle) for all neuroblastoma models. All mice were evaluated based upon surviving complement (measured as percentage remaining) of murine hosts comparing viral-treated tumors with vehicle-treated control tumors starting at day 0 posttreatment. **A,** Evaluation of IMR-32 tumors. Data are depicted through day 66 of the study. **B,** Evaluation of SK-N-AS tumors. Data are depicted through day 40 of the study. **C,** Evaluation of SMS-KAN tumors. Data are depicted through day 101 of the study. **D,** Evaluation of SMS-KANR tumors. Data are depicted through day 52 of the study. The IMR-32 study utilized an *n* = 6 for both vehicle- and Zika-treated cohorts. All other studies utilized an *n* = 10 for both vehicle- and Zika-treated cohorts.

Although our data validate the permissivity of human neuroblastoma tumors to ZIKV infection and correlate this infection with tumor cell death, this does not confirm that there is a survival advantage to the use of ZIKV treatment in the murine host. Therefore, we performed further *in vivo* modeling of human neuroblastoma tumors using IMR-32 cells to screen for indications of survival advantage. Tumors were treated with either ZIKV or vehicle once they reached an average size of approximately 300 mm^3^ and were measured periodically for tumor size (Fig. 6A; Supplementary Fig. S7A). The results revealed that at 28 days posttreatment, all vehicle-treated hosts had exited the study (due to tumor sizes in excess of 2,000 mm^3^), whereas the average remaining tumor size of the treated cohort was approximately 32 mm^3^. After 7 additional days, none of these tumors showed any significant growth, offering a final survival advantage of 100% over PBS-treated controls.

An *in vivo* comparison with SK-N-AS neuroblastoma tumors showed similar results (Fig. 6B; Supplementary Fig. S7B). By 19 days posttreatment, all vehicle-treated hosts had exited the study with tumor masses exceeding 2,000 mm^3^, whereas the ZIKV-treated cohort retained masses with an average size of only approximately 50 mm^3^. Seven days after this timepoint, no significant growth was observed in the treated tumors, conferring a final survival advantage of 100% compared with the vehicle-treated cohort.

Finally, *in vivo* modeling was performed on the matching cell lines, SMS-KAN and SMS-KANR, to determine whether differences could be identified between pretreatment and recurrent tumors with regard to ZIKV therapy. SMS-KAN vehicle-treated tumors all exited their study, with tumor masses exceeding 2,000 mm^3^ by 66 days post-ZIKV treatment (Fig. 6C; Supplementary Fig. S7C). The treated cohort contained a single tumor that exited 14 days posttreatment, although the remaining masses averaged only approximately 24 mm^3^ by the end of the study. By day 108, no recurring masses could be detected, offering a final survival advantage of 90% compared with the vehicle-treated cohort. In comparison, SMS-KANR vehicle-treated tumors all exited their study more rapidly, completing by 35 days posttreatment (Fig. 6D; Supplementary Fig. S7D). None of the ZIKV-treated cohorts contained measurable masses at this timepoint (their tumors had leveled out 11 days earlier than the vehicle-treated cohort). This suggests a 100% survival advantage compared with vehicle-treated SMS-KANR tumors. More importantly, these data indicate that recurrent tumors had no special resistance to ZIKV therapy compared with matching pretreatment tumors.

In addition, qRT-PCR of the resected vehicle-treated tumors confirmed that CD24 expression in the tumor models was comparable to that observed previously between *in vitro* and *in vivo* tumors in this study (Supplementary Figs. S8A–S8D).

Together, these data indicate that ZIKV therapy offers a remarkable survival advantage across an array of neuroblastoma tumors, regardless of MYCN amplification or status as pretreatment versus posttreatment recurrent cancer.

## Discussion

The selective oncolytic effects of ZIKV have been previously demonstrated in various human CNS tumors (33, 50). These studies have revealed that the oncolytic outcomes witnessed are not generalized, even indicating a differential sensitivity between CNS cancers. Zhu and colleagues indicated that ZIKV preferentially killed glioblastoma stem cells as opposed to differentiated tumors or normal neuronal cells, whereas West Nile virus, another neurotropic flavivirus, eliminated both tumor and normal neural cells (30). Correlative studies have proposed the involvement of T-cell infiltration and recruitment to explain ZIKV specificity, although these claims do not explain the differential sensitivity to viral killing observed *in vitro* or in T cell–defective animal models (51, 52). Kaid and colleagues indicated that the modulation of Wnt/β-catenin signaling in ATRT cells correlated with ZIKV-mediated cytotoxicity, although this result was mixed in medulloblastoma models (33). Smith and colleagues further identified a connection between the Wnt signaling pathway and the regulation of the IFN response in various flavivirus infections, although this was performed in HeLa cells, not in cells with a CNS background (53). Our previous work and that of collaborators identified a connection in neuroblastomas between CD24 expression, ZIKV specificity, and the regulation of intracellular antiviral pathways such as type I IFN, NFκB, and Ras (36, 49). However, these results have not been confirmed *in vivo*. Together, these studies offer insights into the possible oncolytic targeting mechanisms of ZIKV but offer no comprehensive prognostics for improved outcomes in a preclinical host.

Here, we report the characterization of the oncolytic effects of ZIKV (strain MR766) on neuroblastoma tumors. Our approach utilized an intratumoral method of introduction, as we envision this therapy to be applied subsequent to the initial surgery step following induction chemotherapy, administered within the tumor bed and margins. This approach was employed in our study to optimally deliver the viral dose locally, with the added benefit of initially minimizing the off-targeting of the application to unintended tissues and organs. The results of this method in our studies demonstrated the elimination of tumor masses in both high-risk MYCN-amplified and non-amplified tumors as well as in matching pretreatment primary and posttreatment recurrent isolates. Furthermore, a human *ex vivo* resection from the posterior mediastinum responded accordingly. In all cases, permissive infections led to a rapid loss of tumor mass, revealing no recurrence even up to 4 weeks posttreatment, offering a remarkable survival advantage to the host. In addition, no side effects, such as conjunctivitis, ataxia, or behavioral abnormalities, were detected in any of the experimental animal subjects from viral application as much as 10 weeks after administration.

These results correlate with the discovery of the association between CD24 expression and ZIKV sensitivity (36). Although we know that CD24 is expressed on a variety of cell types including hematopoietic cells, neural cells, and epithelial stem cells, the presence of CD24 appears to be limited to those cells in a differentiating state and its expression is abandoned upon terminal differentiation (38, 43). Therefore, at any one time, the number of differentiating cells in a host is limited. Likewise, murine and human CD24 are considered orthologous, demonstrating similar functions and expression patterns (37, 54–56). We consider that this may be a contributing factor of the clinical presentation of ZIKV, which yielded no symptoms in the mouse hosts and remains 80% asymptomatic in humans, with the remainder suffering only a mild clinical course (26).

That being said, the expression of CD24 was found to be robust in all of the neuroblastoma tumors. This is not surprising given that neuroblastomas represent neuroblasts which never terminally differentiated and instead continued to grow and divide (57). Because this condition precluded terminal differentiation, the neuroblastomas may very likely have retained the expression of CD24. CD24 is also broadly documented as a driver of tumor proliferation and a predictor of poor prognosis in a variety of cancers, including gastric, colorectal, cervical, breast, and prostate cancers (58–62). This phenotype was demonstrated in SK-N-AS tumors, whose poor expression of CD24 was previously documented *in vitro* but yielded a dramatic increase *in vivo*, suggesting that the upregulation of CD24 offered a selective advantage for tumor growth (36). In our experiments with ZIKV, we identified an alternative role for CD24 as a predictor of neuroblastoma permissiveness. The poor expression of CD24 *in vitro* offered resistance to viral killing, whereas the upregulation of CD24 *in vivo* introduced a dramatic sensitivity to ZIKV killing, correlating with previous results seen *in vitro* when CD24 expression was artificially increased via exogenous expression.

We further addressed the relevance of CD24 in neuroblastoma by modulating its expression *in vivo*. The addition of exogenous CD24 to SK-N-AS (SK-N-AS/CD24) tumors dramatically increased their growth rates, nearly doubling their mass over the same period of time compared with both WT and controls. Likewise, stable knockdown of CD24 in IMR-32 cells (IMR-32/CD24 shRNA) led to a total loss of tumorigenicity. These observations corroborate the earlier supposition that CD24 may offer a selective advantage if not drive an essential function necessary for tumor growth. Recent reports have suggested an immunosuppressive role for CD24 in tumor cells. Barkal and colleagues demonstrated that CD24 can play an antiphagocytic role in tumor cells via interaction with Siglec-10 on tumor-associated macrophages (63). Interestingly, SK-N-AS/CD24 tumors further upregulated CD24 *in vivo* in a manner similar to that observed in both WT and control cells. Despite this, these tumors were not eliminated any faster, indicating that the host induced CD24 upregulation in WT or control tumors was suitable to maximize viral cytotoxicity, whereas the addition of exogenous expression offered a further proliferative advantage exploited by the tumor. This discovery suggests that the upregulation of CD24 in other cancers may indicate viral sensitivity, which could profoundly expand the pool of possible ZIKV therapy targets.

A deeper understanding of the chronology of the treatment process offered additional insights. Shortly following the administration of ZIKV, Zika viral protein NS1 was detected within the tumor, along with high viral copy numbers, correlating with the suppression of tumor growth. This led to a dramatic increase in necrosis within the tissue and the loss of tumor mass, indicating a tumoricidal effect. It is interesting to note that, despite robust viral production within the tumor, viral shedding to the host yielded exceedingly poor titers, on the order of 3 or 4 orders of magnitude less, which diminished with time. Kaid and colleagues reported similar results in other CNS tumors, with cross-infected hosts producing few functional ZIKV particles, suggesting a defect in either viral penetrance or replication in the new host (33). Lazear and colleagues indicated that WT C57BL/6 mice exhibited no morbidity or mortality due to ZIKV infection, whereas *Ifnar1*^−^*^/^*^−^ mice that could not respond to IFNα/β were highly vulnerable to ZIKV infection, losing weight by day 5, and beginning to succumb by day 7 (64). Furthermore, changes in intracellular antiviral pathways were observed by Kedarinath and colleagues after the modulation of CD24 in neuroblastoma cells *in vitro* (49). Together, these data suggest that CD24 may promote an immunomodulatory role in the tumor, presenting a possible explanation for the profound differences observed between tumor and host outcomes *in vivo*.

This study demonstrates the efficacy of ZIKV strain MR766 in solid neuroblastoma tumors by inducing a rapid tumoricidal effect with no evidence of recurrence, regardless of their status as a high-risk MYCN-amplified or posttreatment recurrent tumor. Elimination of the tumor mass in this manner also occurred with no detectable side effects to the host, suggesting a benign alternative therapy or conjunctive treatment to improve the overall outcome. Given the permissive predisposition of ZIKV to CD24-expressing targets, this approach also offers the opportunity to address progenitor cells involved in a broad array of cancers, not only in the pediatric field but among adult CD24-expressing cancers as well.

## Supplementary Material

Supplementary Figure 1 Micro tissue array of human neuroblastoma patient samples stained for CD24.

Supplementary Figure 2 Comparison of the Relative Expression of CD24 across all screened neuroblastoma cells and tumors.

Supplementary Figure 3 Effect of the Zika viral treatment of neuroblastoma tumors on tumor mass over time.

Supplementary Figure 4 Evaluation of the Zika viral time course on neuroblastoma tumors by immunohistochemical staining of CD24.

Supplementary Figure 5 Effect on the change in tumor mass of Zika viral treated CD24-Exogenously expressing SK-N-AS tumors.

Supplementary Figure 6 Direct comparison of the relative expression of CD24.

Supplementary Figure 7 Evaluation of the tumor size of individual neuroblastomas post-treatment with Zika virus over the course of survival studies.

Supplementary Figure 8 The comparison of the relative expression of CD24 across paired neuroblastoma cells and tumors in Kaplan-Meier survival study.
