# Supplementary material for: The Oncolytic Activity of Zika Viral Therapy in Human Neuroblastoma In Vivo Models Confers a Major Survival Advantage in a CD24-dependent Manner
Source: Cancer Res Commun. 2024 Jan 9;4(1):65–80. doi: 10.1158/2767-9764.CRC-23-0221 (PMC10775766; doi:10.1158/2767-9764.CRC-23-0221)
Supplement: Supplementary Figure 1 — Micro tissue array of human neuroblastoma patient samples stained for CD24. [file crc-23-0221-s01.pdf]

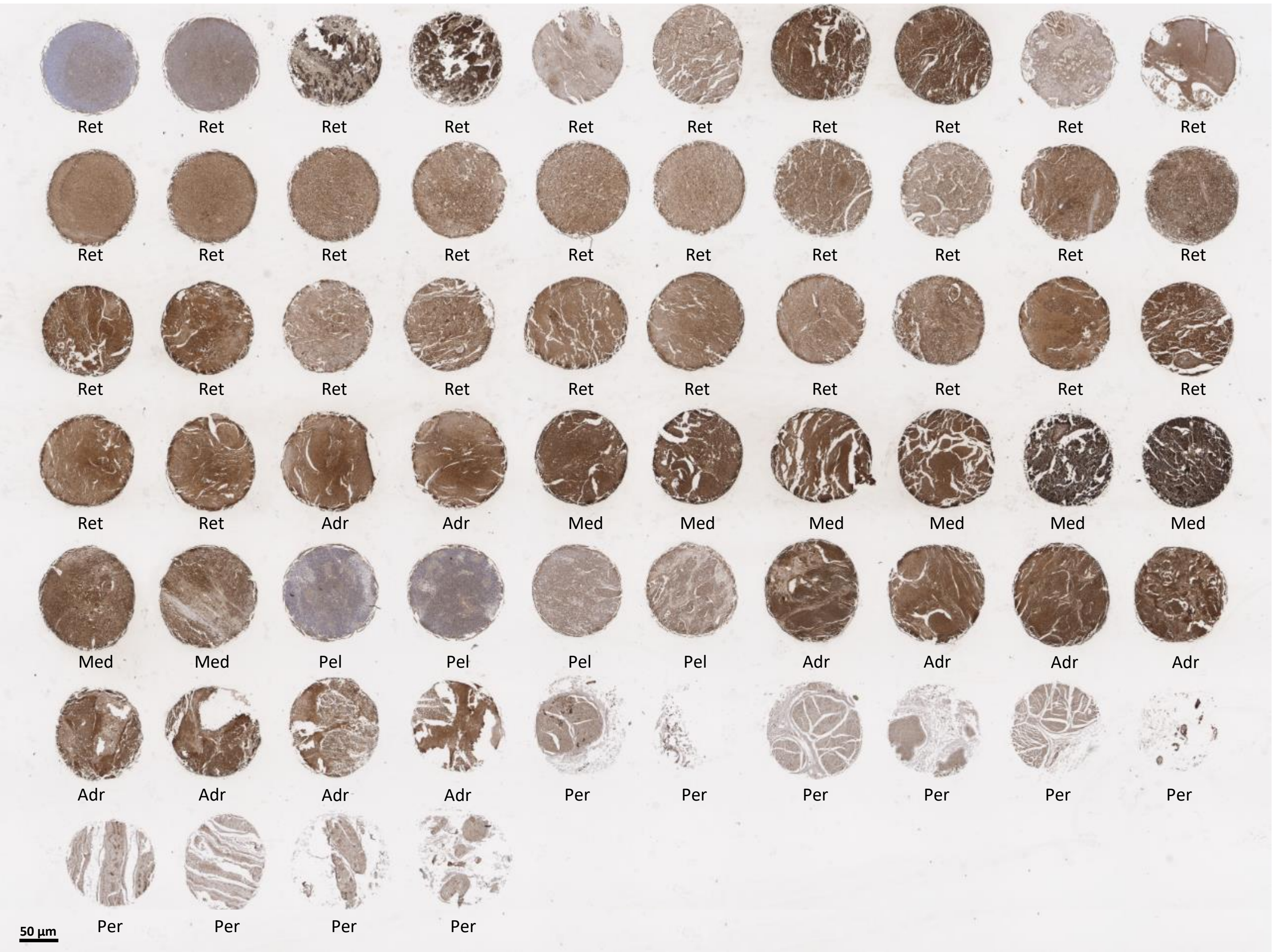

Supplemental Figure 1. Micro tissue array of human neuroblastoma patient samples stained for CD24. Neuroblastoma and peripheral nerve tissue micro array (TMA) slide (NB642c, amsbio), containing 32 cases (in duplicate) was stained by immunohistochemistry for CD24. Tissue samples include tumors isolated from the Adrenal gland (Adr), Mediastinum/left posterior (Med), Pelvic cavity (Pel), and Retroperitoneum (Ret) as well as control Peripheral nerve (Per) tissue. Visualization of the tissues was performed using Keyence and quantified using FIJI ImageJ.
