# Supplementary material for: The Oncolytic Activity of Zika Viral Therapy in Human Neuroblastoma In Vivo Models Confers a Major Survival Advantage in a CD24-dependent Manner
Source: Cancer Res Commun. 2024 Jan 9;4(1):65–80. doi: 10.1158/2767-9764.CRC-23-0221 (PMC10775766; doi:10.1158/2767-9764.CRC-23-0221)
Supplement: Supplementary Figure 2 — Comparison of the Relative Expression of CD24 across all screened neuroblastoma cells and tumors. [file crc-23-0221-s02.pdf]

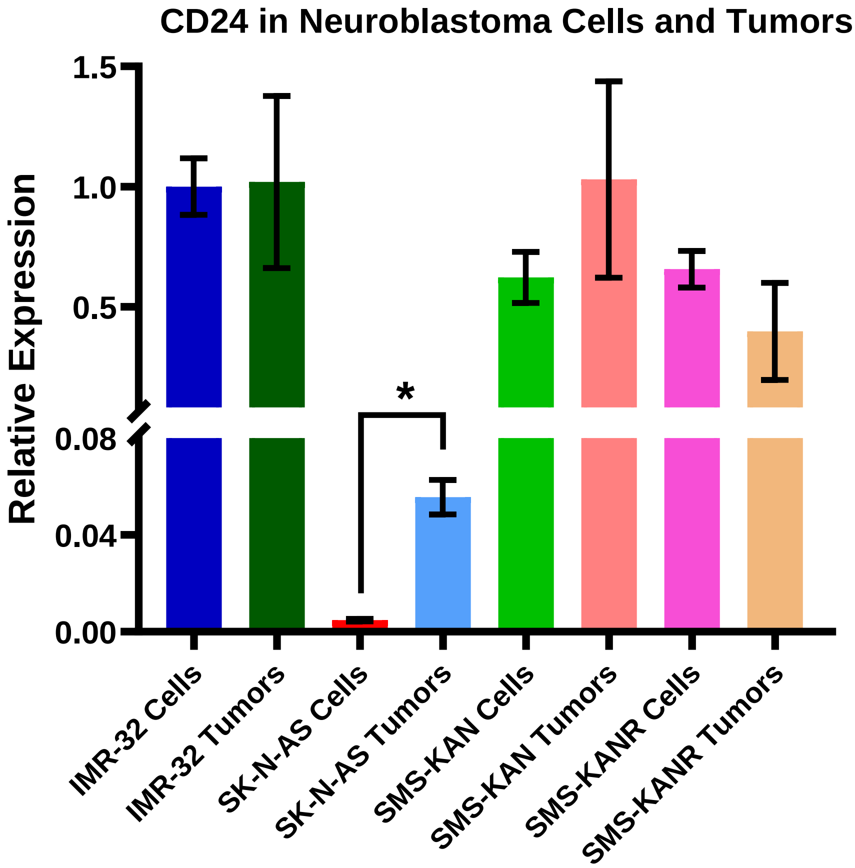

Supplemental Figure 2. Comparison of the Relative Expression of CD24 across all screened neuroblastoma cells and tumors. qRT-PCR was performed comparing IMR-32, SK-N-AS, SMS-KAN, and SMS-KANR cells (*in vitro*, prior to introduction into the mouse host) to tumors (*in vivo*, averaged from post-treatment vehicle treated control tumors) for each neuroblastoma. Expression was normalized to GAPDH. All qPCR data shown are the composite of triplicate wells acquired from triplicate experiments. Error bars represent standard deviation. *p > 0.05 from SK-N-AS Cells, unpaired t-test.
