# Supplementary material for: The Oncolytic Activity of Zika Viral Therapy in Human Neuroblastoma In Vivo Models Confers a Major Survival Advantage in a CD24-dependent Manner
Source: Cancer Res Commun. 2024 Jan 9;4(1):65–80. doi: 10.1158/2767-9764.CRC-23-0221 (PMC10775766; doi:10.1158/2767-9764.CRC-23-0221)
Supplement: Supplementary Figure 3 — Effect of the Zika viral treatment of neuroblastoma tumors on tumor mass over time. [file crc-23-0221-s03.pdf]

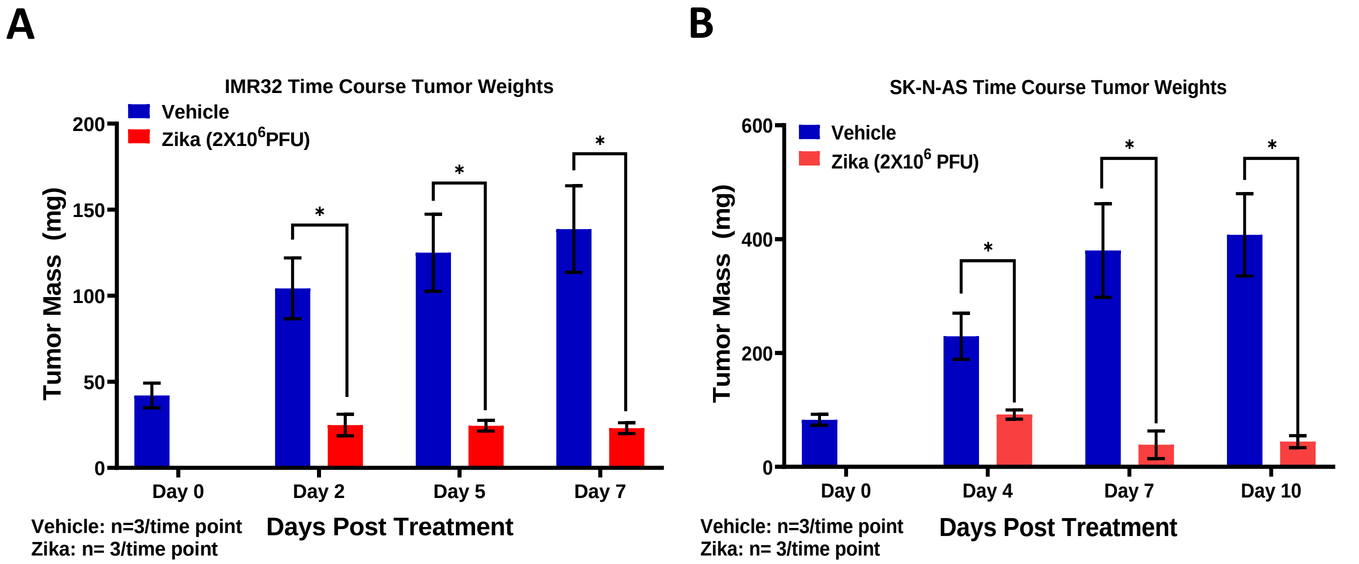

Supplemental Figure 3. Effect of the Zika viral treatment of neuroblastoma tumors on tumor mass over time. ZIKV was introduced once at a concentration of 2 x 10^6 pfu for all tumors. **A)** IMR-32 tumor size was measured at Day 0, 2, 5, and 7 post-treatment compared to vehicle control treatment. Error bars represent standard deviation. *$p < 0.05$ from Vehicle, unpaired t-test, Days 2, 5, & 7. **B)** SK-N-AS tumor size was measured at Day 0, 4, 7, and 10 post-treatment compared to vehicle control treatment. Each time point included n=3 for vehicle and Zika Error bars represent standard deviation. *$p < 0.05$ from Vehicle, unpaired t-test, Days 4, 7, & 10.
