# Supplementary material for: The Oncolytic Activity of Zika Viral Therapy in Human Neuroblastoma In Vivo Models Confers a Major Survival Advantage in a CD24-dependent Manner
Source: Cancer Res Commun. 2024 Jan 9;4(1):65–80. doi: 10.1158/2767-9764.CRC-23-0221 (PMC10775766; doi:10.1158/2767-9764.CRC-23-0221)
Supplement: Supplementary Figure 4 — Evaluation of the Zika viral time course on neuroblastoma tumors by immunohistochemical staining of CD24. [file crc-23-0221-s04.pdf]

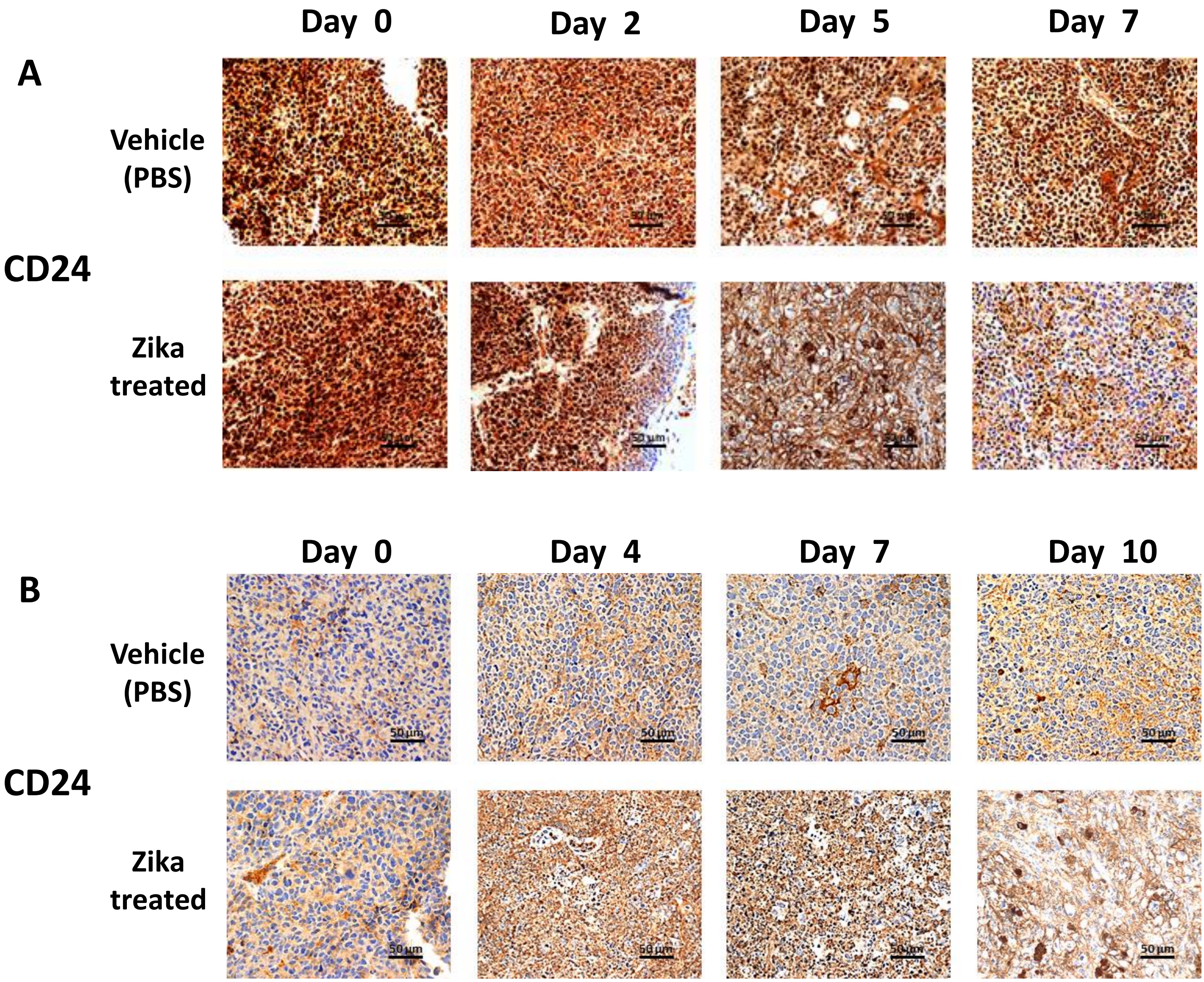

Supplemental Figure 4. Evaluation of the Zika viral time course on neuroblastoma tumors by immunohistochemical staining of CD24. ZIKV treated tumors were compared to vehicle treated tumors by CD24 staining at each time point. **A)** IMR-32 tumors were stained for CD24 at Day 0, 2, 5, and 7 post-treatment. **B)** SK-N-AS tumors were stained for CD24 at Day 0, 4, 7, and 10 post-treatment. Tissues were stained using a Leica Bond Maxfive and images were generated using an Olympus BX43 at a magnification of 40x with a 10x zoom.
