# Supplementary material for: The Oncolytic Activity of Zika Viral Therapy in Human Neuroblastoma In Vivo Models Confers a Major Survival Advantage in a CD24-dependent Manner
Source: Cancer Res Commun. 2024 Jan 9;4(1):65–80. doi: 10.1158/2767-9764.CRC-23-0221 (PMC10775766; doi:10.1158/2767-9764.CRC-23-0221)
Supplement: Supplementary Figure 5 — Effect on the change in tumor mass of Zika viral treated CD24-Exogenously expressing SK-N-AS tumors. [file crc-23-0221-s05.pdf]

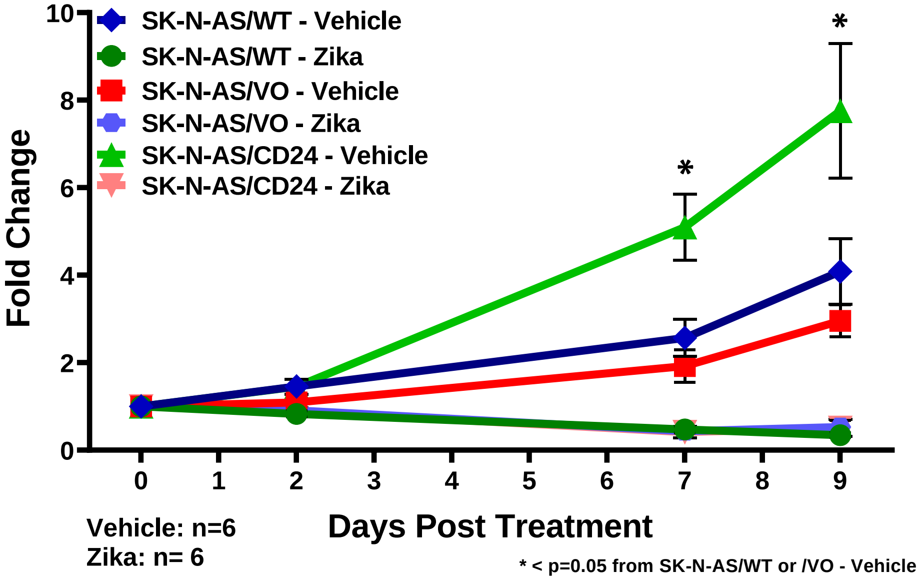

Supplemental Figure 5. Effect on the change in tumor mass of Zika viral treated CD24-Exogenously expressing SK-N-AS tumors. Evaluation of the application of ZIKV to SK-N-AS/WT (Wild Type), SK-N-AS/VO (Vector Only control), and SK-N-AS/CD24 (stable exogenous expression of CD24) tumors in NCr nude mice, measuring for changes in the fold change of tumor mass, comparing viral treated tumors and vehicle treated control tumors to SK-N-AS/WT at Day 0. Virus was introduced once at a concentration of 2 x 10^6 pfu for all tumors. Data is depicted through Day 9 post-treatment. All comparisons utilized an n = 6 for both Vehicle and Zika treated cohorts. Error bars represent standard deviation. *p > 0.05 from SK-N-AS/WT or /VO - Vehicle, one-way ANOVA for Days 7, & 9.
