# Supplementary material for: The Oncolytic Activity of Zika Viral Therapy in Human Neuroblastoma In Vivo Models Confers a Major Survival Advantage in a CD24-dependent Manner
Source: Cancer Res Commun. 2024 Jan 9;4(1):65–80. doi: 10.1158/2767-9764.CRC-23-0221 (PMC10775766; doi:10.1158/2767-9764.CRC-23-0221)
Supplement: Supplementary Figure 6 — Direct comparison of the relative expression of CD24. [file crc-23-0221-s06.pdf]

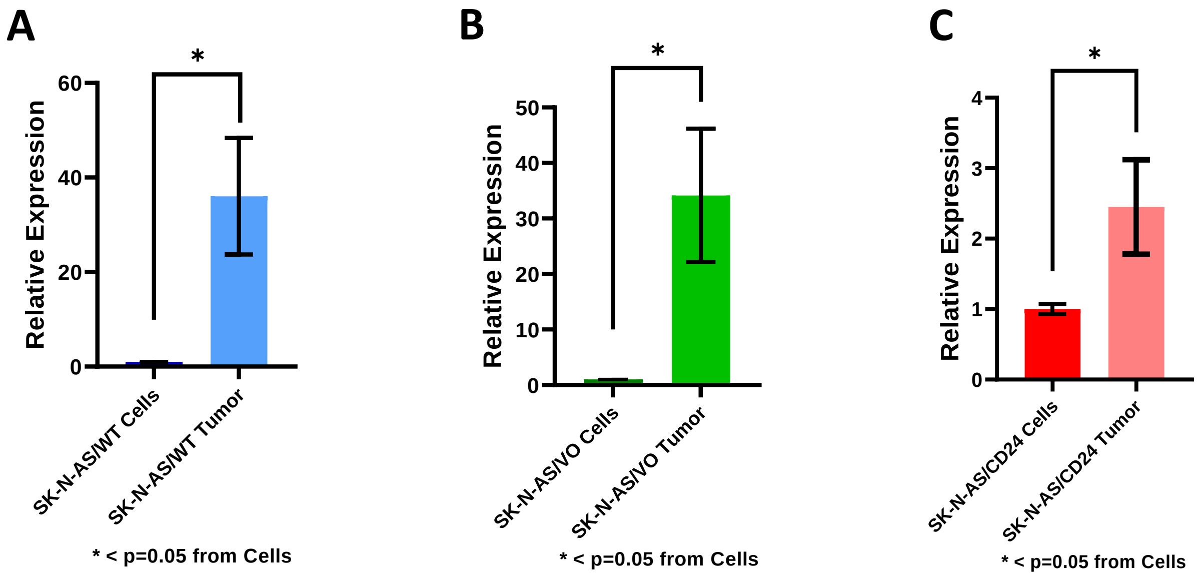

Supplemental Figure 6. Direct comparison of the relative expression of CD24. Expression was measured between cells (*in vitro*) to tumors (*in vivo*) for **A)** SK-N-AS/WT, **B)** SK-N-AS/VO, and **C)** SK-N-AS/CD24. CD24 expression was assessed using qRT-PCR comparing cells prior to injection into murine hosts to tumors resected post-study. All qPCR expression data shown was normalized to GAPDH and are the composite of triplicate wells acquired from triplicate experiments. Error bars represent standard deviation. *p > 0.05 from A) SK-N-AS/WT Cells, B) SK-N-AS/VO Cells, or C) SK-N-AS/CD24 Cells, unpaired t-test for Tumor.
