# Supplementary material for: The Oncolytic Activity of Zika Viral Therapy in Human Neuroblastoma In Vivo Models Confers a Major Survival Advantage in a CD24-dependent Manner
Source: Cancer Res Commun. 2024 Jan 9;4(1):65–80. doi: 10.1158/2767-9764.CRC-23-0221 (PMC10775766; doi:10.1158/2767-9764.CRC-23-0221)
Supplement: Supplementary Figure 7 — Evaluation of the tumor size of individual neuroblastomas post-treatment with Zika virus over the course of survival studies. [file crc-23-0221-s07.pdf]

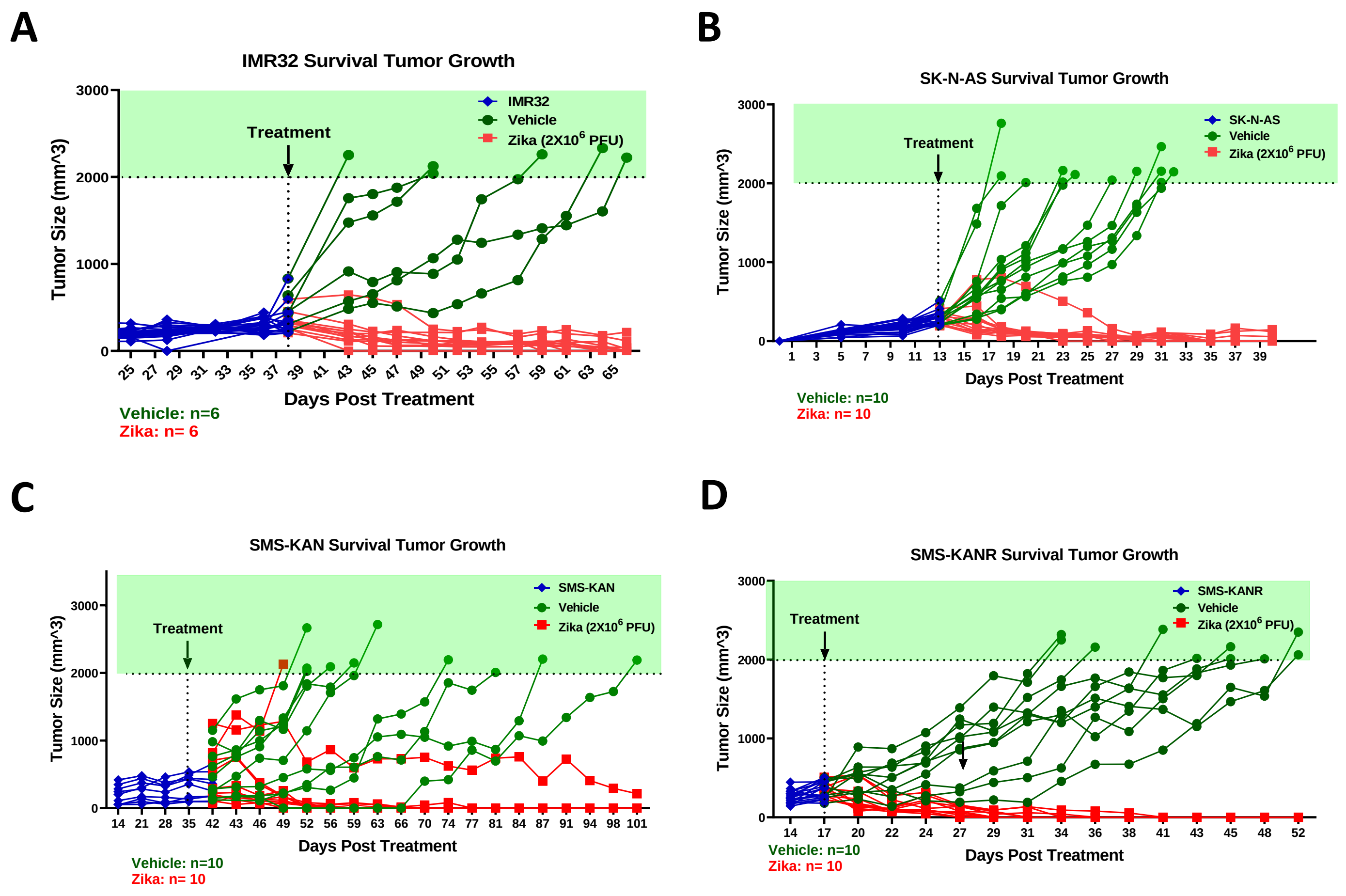

Supplemental Figure 7. Evaluation of the tumor size of individual neuroblastomas post-treatment with Zika virus over the course of survival studies. ZIKV was introduced once at a concentration of 2 x 10^6 pfu (compared to vehicle) for all neuroblastoma models. All mice were evaluated based upon "Tumor Size" (mm^3) in murine hosts comparing viral treated tumors to vehicle treated control tumors starting at Day 0 post-treatment. **A)** Evaluation of IMR-32 tumors. Data is depicted through Day 66 of the study. **B)** Evaluation of SK-N-AS tumors. Data is depicted through Day 40 of the study. **C)** Evaluation of SMS-KAN tumors. Data is depicted through Day 101 of the study. **D)** Evaluation of SMS-KANR tumors. Data is depicted through Day 52 of the study. The IMR-32 study utilized an n = 6 for both Vehicle and Zika treated cohorts. All other studies utilized an n = 10 for both Vehicle and Zika treated cohorts.
