# Supplementary material for: The Oncolytic Activity of Zika Viral Therapy in Human Neuroblastoma In Vivo Models Confers a Major Survival Advantage in a CD24-dependent Manner
Source: Cancer Res Commun. 2024 Jan 9;4(1):65–80. doi: 10.1158/2767-9764.CRC-23-0221 (PMC10775766; doi:10.1158/2767-9764.CRC-23-0221)
Supplement: Supplementary Figure 8 — The comparison of the relative expression of CD24 across paired neuroblastoma cells and tumors in Kaplan-Meier survival study. [file crc-23-0221-s08.pdf]

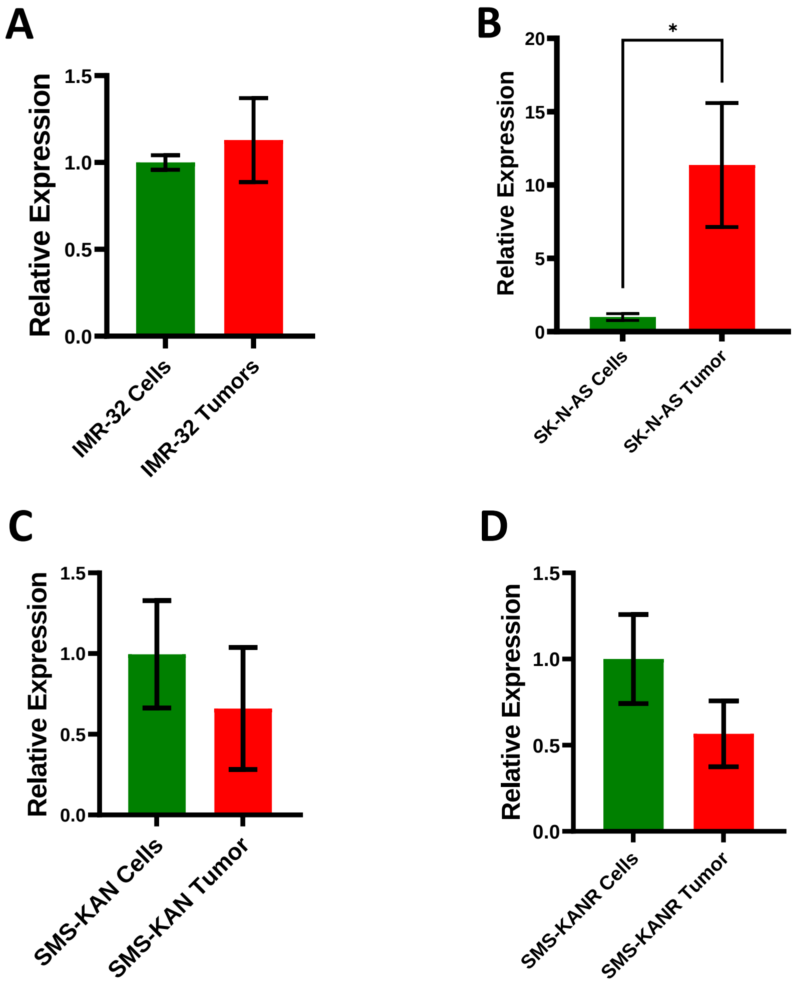

Supplemental Figure 8. The comparison of the relative expression of CD24 across paired neuroblastoma cells and tumors in Kaplan-Meier survival study. Relative expression for CD24 was assessed using qRT-PCR comparing *in vitro* cells prior to injection to *in vivo* samples (averaged from tumors resected from murine hosts post-survival) for each neuroblastoma. Expression was normalized to GAPDH. All qPCR data shown are the composite of triplicate wells acquired from triplicate experiments. Error bars represent standard deviation. *p > 0.05 from SK-N-AS Cells, unpaired t-test.
